# Digital Holographic Microscopy for Label-Free Detection of Leukocyte Alternations Associated with Perioperative Inflammation after Cardiac Surgery

**DOI:** 10.3390/cells11040755

**Published:** 2022-02-21

**Authors:** David Rene Steike, Michael Hessler, Eberhard Korsching, Florian Lehmann, Christina Schmidt, Christian Ertmer, Jürgen Schnekenburger, Hans Theodor Eich, Björn Kemper, Burkhard Greve

**Affiliations:** 1 Department of Radiotherapy-Radiooncology, University Hospital Muenster, Albert-Schweitzer-Campus 1, Building A1, 48149 Muenster, Germany; d.steike@uni-muenster.de (D.R.S.); hans.eich@ukmuenster.de (H.T.E.); 2 Department of Anesthesiology and Intensive Care and Pain Medicine, University Hospital of Muenster, Albert-Schweitzer-Campus 1, 48149 Muenster, Germany; michael.hessler@uni-muenster.de (M.H.); florian.lehmann@ukmuenster.de (F.L.); christina.schmidt@ukmuenster.de (C.S.); christian.ertmer@ukmuenster.de (C.E.); 3 Institute of Bioinformatics, Medical Faculty, University of Muenster, 48149 Muenster, Germany; eberhard.korsching@uni-muenster.de; 4 Biomedical Technology Center, Medical Faculty, University of Muenster, 48149 Muenster, Germany; schnekenburger@uni-muenster.de

**Keywords:** quantitative phase imaging, digital holographic microscopy, flow cytometry, label free, cardiac surgery, CPB, systemic inflammation, epinephrine

## Abstract

In a prospective observational pilot study on patients undergoing elective cardiac surgery with cardiopulmonary bypass, we evaluated label-free quantitative phase imaging (QPI) with digital holographic microscopy (DHM) to describe perioperative inflammation by changes in biophysical cell properties of lymphocytes and monocytes. Blood samples from 25 patients were investigated prior to cardiac surgery and postoperatively at day 1, 3 and 6. Biophysical and morphological cell parameters accessible with DHM, such as cell volume, refractive index, dry mass, and cell shape related form factor, were acquired and compared to common flow cytometric blood cell markers of inflammation and selected routine laboratory parameters. In all examined patients, cardiac surgery induced an acute inflammatory response as indicated by changes in routine laboratory parameters and flow cytometric cell markers. DHM results were associated with routine laboratory and flow cytometric data and correlated with complications in the postoperative course. In a subgroup analysis, patients were classified according to the inflammation related C-reactive protein (CRP) level, treatment with epinephrine and the occurrence of postoperative complications. Patients with regular courses, without epinephrine treatment and with low CRP values showed a postoperative lymphocyte volume increase. In contrast, the group of patients with increased CRP levels indicated an even further enlarged lymphocyte volume, while for the groups of epinephrine treated patients and patients with complicative courses, no postoperative lymphocyte volume changes were detected. In summary, the study demonstrates the capability of DHM to describe biophysical cell parameters of perioperative lymphocytes and monocytes changes in cardiac surgery patients. The pattern of correlations between biophysical DHM data and laboratory parameters, flow cytometric cell markers, and the postoperative course exemplify DHM as a promising diagnostic tool for a characterization of inflammatory processes and course of disease.

## 1. Introduction

Perioperative inflammation is common in patients undergoing cardiac surgery with cardiopulmonary bypass and mainly originates from the surgical trauma, ischemia-reperfusion injury and contact activation of cells with the surface of the cardiopulmonary bypass [1,2,3]. Systemic inflammation can vary significantly between patients [1,3]. The cause of systemic inflammation after cardiac surgery is multifactorial and some predisposing factors have already been described, such as age or comorbidities [2,3]. Moreover, it was reported that individuals with profound systemic inflammation after surgery show an increased risk of adverse outcomes [4,5,6,7].

Within the course of inflammation, circulating leukocytes undergo significant changes including morphology, expression of surface antigens, cell mechanics (e.g., deformability) and mobility [8,9,10,11,12]. Currently, marker-based flow cytometry (FCM) represents the gold standard to investigate and describe blood cell differentiation and subset content but also bears several challenges and uncertainties [13]. The latter are related to the involved complex multiparametric measuring and laborious staining protocols of red blood cell lysis. Especially, the large variety of inflammation related markers and their complex interdependence usually hinders the usage of single markers for reliable prediction and in sum can lead to misjudgments [13,14,15].

To address these challenges, within recent years various label-free methods for the chemical and physical characterization of cells and tissues were developed and evaluated [16,17].

Quantitative phase imaging (QPI) is an emerging technique for minimally invasive analysis of almost transparent biological specimens, based on the detection of sample-induced optical path length changes against the surrounding environment, such as buffer or embedding media [18]. Digital holographic microscopy (DHM) [19] is an interferometry-based variant of QPI. DHM QPI images provide access to biophysical properties of cells such as refractive index, dry mass, volume and morphology related parameters, which are connected to different physiological features and functions [20,21,22]. Earlier studies demonstrated the potential of DHM in various applications areas. These, for example, include the analysis of blood [23], endothelial [24] and neuronal cells [25] or cancer cell phenotyping [26]. Moreover, utilization of DHM for label-free quantification of infections on single cell scale [27], cell culture quality control [28], the quantification of the cellular response to drugs and toxins [29,30] as well as the visualization of chromosome segregation [31], were reported. In addition, in recent studies DHM was applied to assess inflamed colonic segments and fibrotic remodeling of stenotic colon tissue areas [32,33]. These results indicate DHM as a promising tool for tracking inflammatory processes on a cellular scale.

Based on this context, the aim of this prospective exploratory pilot study is the evaluation of the capabilities of DHM for marker free detection and description of inflammation-induced changes in biophysical cell parameters of peripheral blood mononuclear cells (PBMCs). Investigations were performed on a collective of 25 patients undergoing elective cardiac surgery with cardiopulmonary bypass. In detail, we isolated and manually measured with DHM about 30,000 monocytes and lymphocytes isolated from blood samples taken perioperatively on different days. The retrieved DHM QPI images were subsequently evaluated for changes in refractive index, volume, dry mass and cell shape related form factor and correlated with state-of-the-art flow cytometric data and clinical parameters.

## 2. Materials and Methods

### 2.1. Study Design and Blood Collection

**Figure 1:**
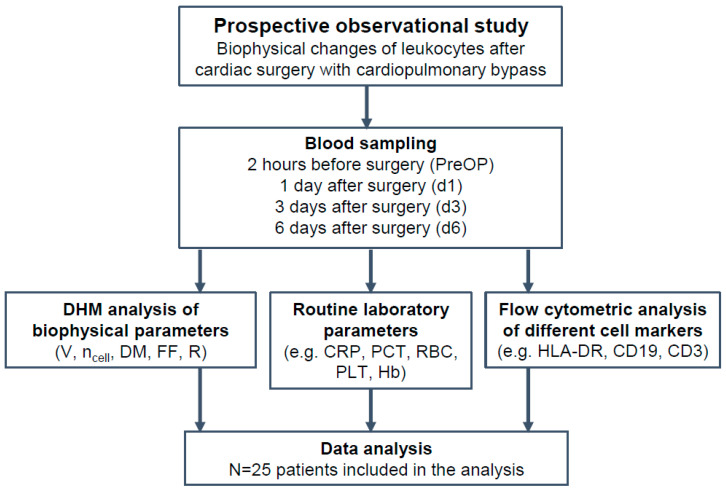
Overview of the study. Biophysical cell parameters acquired by DHM: Volume (V), Refractive index (*n*_cell_), Dry Mass (DM), Form Factor (FF), Radius (R), Routine laboratory parameters: Leukocytes, C-reactive protein (CRP), Procalcitonin (PCT), Red Blood Cells (RBC), PLT (platelets), Hemoglobin (Hb), Flow cytometric surface markers (HLA-DR, CD19, CD3) as shown in Table S2 in Supplement S1 file.

The study was approved by the local ethics committee of the University Hospital (Münster, Germany) and Medical Association Westphalia-Lippe (registration number 2017-240-f-S). It was designed as a prospective observational pilot study (Figure 1). Written informed consent was obtained from 25 patients (age ≥ 18 years) scheduled for elective cardiac surgery with cardiopulmonary bypass of ischemic heart disease, or to be operated on one or more heart valves. Patients with serious comorbidities or acute preoperative illness such as an acute infection or pneumonia were excluded to avoid confounding. The occurrence of serious complications (e.g., organ dysfunctions such as acute kidney injury or development of infections) and the need for ionotropic support with epinephrine, as a surrogate for circulatory failure, was noted for each individual patient. Table S1 in Supplement S1 file lists complications of all patients included in the study.

Patient blood samples were collected within two hours before surgery (PreOP) and at day 1 (d1: 22–26 h after surgery), at day 3 (d3: approx. 72 h) and at day 6 (d6: approx. 144 h) after the operation. Blood samples were obtained from inlaying peripheral catheters. 

Perioperative blood samples were analyzed using DHM and flow cytometry to detect inflammatory processes as described below. Based on earlier experiences in quantification of inflammation with DHM [34] as well as on promising results from the literature [9,35], we focused our research on PBMCs which particularly consist of lymphocytes (T cells, B cells, natural killer cells) and monocytes. In order to meet strict preanalytical standards for flow cytometric analysis [13], EDTA blood was analyzed directly after blood withdrawal. Additionally, routine laboratory parameters, e.g., C-reactive protein (CRP), procalcitonin, liver enzymes, red blood cell count (RBC), hemoglobin concentration (Hb), and platelet counts, were determined in the central laboratory of the university hospital. 

### 2.2. Isolation of Peripheral Blood Mononuclear Cells for DHM Analysis

PBMCs were isolated by mixing 6 mL whole blood with 24 mL PBS/2 mM EDTA. Afterwards, 13 mL Ficoll separation solution was layered and centrifuged at 400× *g* for 35 min without using brake. The isolated PBMC layer was carefully removed and washed twice with 30 mL PBS/2 mM EDTA buffer. The supernatant was completely discarded. After transferring the cell solution through a 30 µL filter to remove larger impurities, cells were centrifuged for 5 min at 100× *g* and finally resuspended in 2.5 mL buffer solution. 

Isolation of monocytes: To modify as few cells as possible in the preparation process for DHM investigations, monocytes were isolated from PBMCs in a negative selection by utilizing magnetic beads of an isolation kit (human Pan Monocyte Isolation Kit, Miltenyi Biotec, Bergisch Gladbach, Germany) following the instructions of the manufacturer. In detail, 3 × 10^6^ cells were centrifuged at 300× *g* for 10 min and subsequently resuspended in 40 µL isolation buffer. Then 10 µL blocking reagent and 10 µL Biotin antibody cocktail was added. After 15 min incubation at 4 °C, 30 µL isolation buffer and 20 µL Anti-Biotin MicroBeads were added and incubated again for 15 min. Cells were pelleted at 300× *g* for 10 min and dissolved in 390 μL buffer solution. The cells were then applied onto a magnetic activated cell-sorting (MACS) column (MS columns, Miltenyi Biotec) and washed three times with 500 µL buffer. The flow-through fraction contained the unlabeled enriched monocytes, which were stored on ice before starting the subsequent digital holographic measurement.

Isolation of lymphocytes: PBMCs were first washed with isolation buffer to remove remaining platelets and centrifuged by 300× *g* for 10 min. The cell pellet was resuspended in 80 µL buffer solution and mixed with 20 µL of CD14-MicroBeads followed by 15 min incubation at 4 °C. After removal of CD14 positive monocytes, the unlabeled lymphocytes reside in the flow-through and were washed three times with 500 µL isolation buffer. 

For DHM measurements about 1 × 10^5^ cells were diluted with 1 mL PBS/2 mM EDTA and transferred into 35 mm µ-dishes (Ibidi, Martiensried, Germany). At each time point per patient, 150 lymphocytes and 150 monocytes were measured. Since monocytes quickly settle and bind to the surface, and therefore change their structure [36], the µ-dish bottom was coated with a small amount of a stiff matrix (Matrigel, Corning, Kaiserslautern, Germany) that prevents adhesion of monocytes and also prevents fixation or manipulation during measurement [37]. 

### 2.3. Quantitative Phase Imaging with Digital Holographic Microscopy

**Figure 2:**
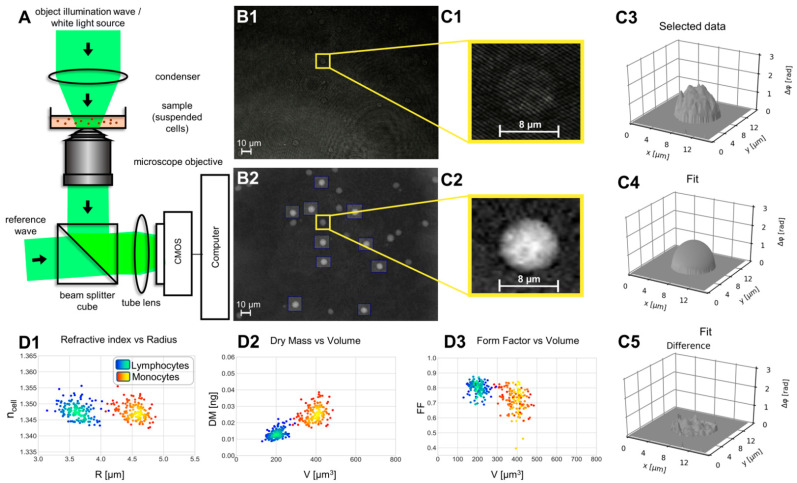
Measurement concept and strategy for retrieval of biophysical parameters from QPI images of lymphocytes and monocytes. (**A**) Sketch of the utilized off-axis DHM microscope (laser light wavelength: 532 nm). (**B1**) Representative digital off-axis hologram of suspended lymphocytes; (**B2**) DHM QPI image reconstructed numerically from the hologram. Cells marked with boxes were manually selected for a further evaluation for retrieval of cell radius R and integral cellular refractive index *n*_cel_. (**C1**) Enlarged area of the hologram (**B1**) with an included lymphocyte that illustrates the holographic off-axis carrier fringe pattern. (**C2**) Enlarged area of the DHM QPI image (**B2**) with an included lymphocyte. (**C3**) Pseudo three-dimensional plot of the segmented phase data in (**C2**,**C4**): Two-dimensional fit to the phase data in (**C3**) during the numerical procedure for retrieval of R and *n*_cell_ [28]. (**C5**) The difference of the phase data in (**C3**,**C4**) was used to validate the two-dimensional fitting process. (**D1**) Representative scatterplot of *n*_cell_ vs. R retrieved from 150 lymphocytes and 150 monocytes from the preoperative blood sample of an individual patient measured six days after surgery. (**D2**) Scatterplot of the corresponding dry mass DM vs. cell volume *V*. (**D3**) Scatterplot of the corresponding cell shape related form factor FF vs. V.

For QPI of lymphocytes and monocytes an inverted Nikon Ts2R microscope (Nikon, Tokyo, Japan) equipped with an attached fiber optic Mach-Zehnder interferometer off-axis DHM module (Figure 2A) and a motorized microscope stage (Märzhäuser, Wetzlar, Germany) based on previously described concepts [38,39] was applied. The coherent light source for the recording of digital holograms was a fiber coupled solid state laser (Cobolt 06-DPL, λ = 532 nm, Cobolt AB, Solna, Sweden). Suspended cells, prepared as described in Section 2.2, were observed in petri dishes (ibidi µ-Dish ibidi GmbH, Munich, Germany). The sample was illuminated with laser light in transmission (object wave). Digital off-axis holograms (Figure 2B1) of manually selected cells were recorded with a complementary metal-oxide-semiconductor (CMOS) sensor (UI-3260CP-M-GL, IDS Imaging Development Systems GmbH, Obersulm, Germany) using a 40× microscope lens (Nikon CFI Plan Achromat 70×/0.4, Nikon, Tokyo, Japan). All experiments were performed at room temperature and normal atmosphere. The reconstruction of the acquired digital holograms and optional numerical refocusing was performed with previously reported algorithms [26,40] utilizing custom built software, implemented in Python 3.7. The resulting DHM QPI images (Figure 2B2) quantify the optical path length delay caused by the investigated cells to the surrounding buffer medium. The cell induced quantitative phase contrast Δ*φ*_cell_ depends on the cell thickness *d*, the integral cellular refractive index *n*_cell_, the refractive index *n*_medium_ of the buffer medium and the wavelength *λ* of the laser light used in the DHM system [26,41]:

(1) $$\Delta \varphi_{\mathrm{cell}}\left(x,y\right)=\left(2\pi /\lambda \right)\cdot d\left(x,y\right)\cdot \left(n_{\mathrm{cell}}-n_{\mathrm{medium}}\right)$$

### 2.4. Evaluation of DHM QPI Images for Determination of Biophysical Parameters and Morphology Changes

Subsequent evaluation of DHM QPI images enables the retrieval of biophysical cell parameters such as volume V, integral cellular refractive index *n*_cell_, and dry mass DM that are related to various cellular features and processes [20,21,22]. Therefore, in this study *n*_cell_, V and DM of isolated lymphocytes and monocytes were determined preoperatively (PreOP), as well as subsequently on day 1 (d1), day 3 (d3) and day 6 (d6) using custom build software, implemented in Python 3.7. For each sample, with the setup in Figure 2A, digital holograms of *N* = 150 selected cells with spherical appearance were recorded (Figure 2B1,C1). Obviously damaged cells and attached cells showing deformations were not considered in the further QPI image evaluation. Monocytes that were already tethered by platelets in form of platelet–monocyte complexes (PMCs) were also excluded due to a non-spherical shape (for illustration see Figure S1 in Supplement S1 file). From the numerically reconstructed DHM QPI images of individual single cells (Figure 2B2,C2), in an initial step, the integral cellular refractive index *n*_cell_, which quantifies the cell density, and is directly related to the intracellular solute concentration [42], as well as the cell radius R, were determined. To retrieve the two unknown parameters cell refractive index and radius in Equation (1) for each selected single cell a two-dimensional numerical fitting procedure was applied as illustrated in Figure 2C3–C5. Therefore, as described with details in [28], the cell thickness d(x,y) in Equation (1) was estimated by the sphere function. The assumption of the sphere model allows retrieval and decoupling of *n*_cell_ and R iteratively by fitting of Equation (1) to the measured phase data based on the Gauss-Newton method, considering existing knowledge of the image scale (determined by calibration with an object micrometer) and the refractive index of the buffer medium (*n*_medium_ = 1.337, measured with an Abbe refractometer). The scatterplot in Figure 2D1 illustrates the resulting data clouds *n*_cell_ vs. R obtained from 150 lymphocytes and 150 monocytes during a single preoperative measurement of an individual patient measured six days after surgery. In addition, from the parameter R the cell volume V = (4/3)π*R*^3^ was calculated. Subsequently, as described in [28] from the parameters V, *n*_cell_ and *n*_medium_, the cellular dry mass DM = (V/α) (*n*_cell_-*n*_medium_) was determined assuming a refractive index increment of α = 0.2 mL/g [42,43]. Figure 2D2 depicts a corresponding scatter plot of DM vs. V. Moreover, to quantify shape changes of the cells, with respect to a spherical appearance, the projected area A and the perimeter P of the investigated single cells were determined from segmented DHM QPI images as shown in Figure 2C4 and then used to calculate the form factor FF = 4πA/P^2^ [44] with FF ∈ [0, 1]. For ideal spherical cells, the projected area *A* corresponds to a circle for which FF is maximum *(FF_max_* = 1).

### 2.5. Flow Cytometric Analyses

**Figure 3:**
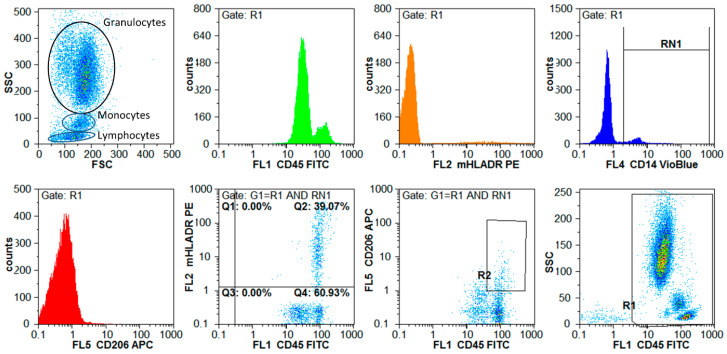
Measuring and gating strategy for four color flow cytometric analysis of a patient lysed whole blood sample. In the forward scatter (FSC, cell size) and side scatter (SSC, granular structure) dot plot the different cellular components of the whole blood could be assigned, where the monocytes appear slightly larger than the lymphocytes. Leucocytes were identified in the lower right plot (FL1 CD45 against SSC) by setting a region-gate around the CD45 positive cells (pan-leucocyte gate R1). CD14 positive monocytes were gated (RN1) in the upper right histogram by setting a backgate on pan leucocytes (R1). Both monocyte gate (RN1) and pan leucocytes gate (R1) were combined and used as backgate to quantify the HLA-DR positive monocytes, in the dot plot of FL1 CD45 against FL2 mHLA-DR. HLA-DR positive monocytes appear in Q2 of the quadrant gate. For the quantification of CD206 positive cells, the same backgate (R1 and RN1) was used as illustrated in dot plot FL1 CD45 against FL5 CD206 and CD206 positive monocytes were quantified in the gate R2. This procedure allowed quantification of CD45 positive leucocytes, CD14 positive monocytes, HLA-DR positive monocytes and CD206 positive monocytes.

Flow cytometric marker analysis was conducted as previously described [14] to identify and quantify leucocyte subsets by their surface marker expression, which were described as relevant for inflammatory processes. Briefly, 100 µL whole blood was incubated with 10 µL of each marker specific antibody for 15 min at room temperature in the dark (detailed information on measured cell markers and antibodies are given in Tables S2 and S3 in Supplement S1 file). Next, 900 µL erythrocyte lysing reagent (BD Pharm Lyse, BD Biosciences, Heidelberg, Germany) was added, the solution incubated for 20 min in the dark and diluted by addition of 1 mL PBS/2 mM EDTA and 0.5% BSA. Lysed blood samples were centrifuged at 400× *g* for 10 min and afterwards resuspended into 1 mL PBS/2 mM EDTA/0.5% BSA. Measurements were performed immediately by a flow cytometer (Cyflow Space, Sysmex/Partec, Görlitz, Germany). Excitation was performed at 375 nm (UV laser), 488 nm (argon laser), and 638 nm (laser diode). Data were acquired, visualized, and gated using FloMax software (Quantum Analysis, Münster, Germany). Figure 3 illustrates the data acquisition and gating strategy of a four-color measurement by representative data. Isolated PBMCs were used to quantify apoptotic/necrotic cells by using Annexin V-FITC Apoptosis detection kit (BD Biosciences). In detail, 1 × 10^5^ PBMCs were suspended in 100 µL apoptosis binding buffer, 5 µL Annexin V and 5 µL propidium iodide were added to the cell suspension and incubated for 15 min in the dark. After final addition of binding, buffer cells were measured by flow cytometry using 488 nm Argon-laser for excitation. Fluorescence emission was measured at 525 nm in FL1 (FITC) and 675 nm in FL3 (propidium iodide).

### 2.6. Statistical Analyses and Outcome Measures

General descriptive statistics were calculated using IBM SPSS Statistics 24 (IBM Corporation, Somers, NY, USA) and GraphPad Prism 9.1 (GraphPad Software, San Diego, CA, USA). Boxplots and further graphical representations were created with GraphPad Prism 9.1, OriginPro 2021b (OriginLab Corporation, Northampton, MA, USA), R platform version 4.1.2, and Python 3.7 (WinPython64-3771 utilizing matplotlib 3.2.1).

Two-sided Pearson correlation tests (R platform version 4.1.2) and the corresponding coefficients were used to compare differences of parameters between single measurement days. Scatter plots (including a simple linear regression) were additionally employed here to monitor the individual sample behavior of all sample cohort members. The R code for these procedures is available on reasonable request. 

Additionally, a bootstrap procedure was implemented in R to analyze whether differences of the DHM biophysical parameters showed stable effects [45]. Therefore, values were sampled with replacement (on average 36.7%). In all cases the sampling number was set to 10,000 which resulted in stable sample estimates. For all DHM data sets measurement entities were sampled independently. The sample *p* value was calculated based on the number of samples that missed the range of the original mean values and their corresponding standard deviations (threshold level: 0.3 SD). All sample *p* values were adjusted utilizing the Benjamini-Hochberg procedure to determine the multiple testing error.

The statistical significance level was set to 0.05 for all analyses. Inferential statistics are intended to be exploratory (i.e., as a basis for hypotheses), rather than confirmatory, and are interpreted accordingly. The comparison-wise type-I error rate is controlled instead of the experiment-wise error rate.

## 3. Results

### 3.1. The Selected Patient Cohort Had a Typical Spectrum of Features for Cardiac Surgery

The study population (*N* = 25) consisted of 16 male patients (64%) and nine female patients (36%). The mean age of the patients was 67 ± 15 years. Detailed patient characteristics are provided in Supplementary Table S1. About 50% of the patients received coronary artery bypass grafting (CABG, 12 of 25), 28% aortic valve replacement (7 of 25) and 20% had a mitral valve reconstruction (5 of 25). Four patients received a combination of CABG and heart valve surgery. Two patients died within 24 h after surgery because of myocardial pump failure. Three patients left the hospital before the fourth blood draw on day 6 due to a complication-free course; in these cases no blood sample could be drawn. 

Patients were assigned to different groups according to the postoperative course. A fraction of 16 from 25 patients (68%) showed a postoperative course without any complications and were extubated at the day of surgery (hereinafter referred to as regular course). In contrast, nine patients (32%) developed severe complications in the postoperative period. These complications include cardiopulmonary resuscitation because of myocardial infarction, acute kidney injury and pneumonia. Patients suffering from complications are, in the following text, referred to as complicated course (detailed information about complications are provided in Table S1 in Supplement S1 file). Another eight patients were considered separately as they received epinephrine intra- and postoperatively as ionotropic support to stabilize the cardiovascular system. Moreover, in seven patients, there was a noticeably significant postoperative increase in CRP, most likely an expression of inflammation following surgical trauma rather than infection. Therefore, data from patients with a profound increase in CRP was considered separately and compared to patients with modest CRP increase using a cut-off value of 14 mg/dL [46].

### 3.2. Biophysical Parameters Allow Clear Cell Differentiation between Lymphocytes and Monocytes and Increase in Scattering Immediately after Surgery

A set of 200 blood samples from 25 patients, acquired preoperatively and on day 1, day 3 and day 6 after surgery, were analyzed with DHM. Within these samples 30,000 QPI images of manually identified PBMCs were evaluated for biophysical parameters as described in Section 2.4.

**Figure 4:**
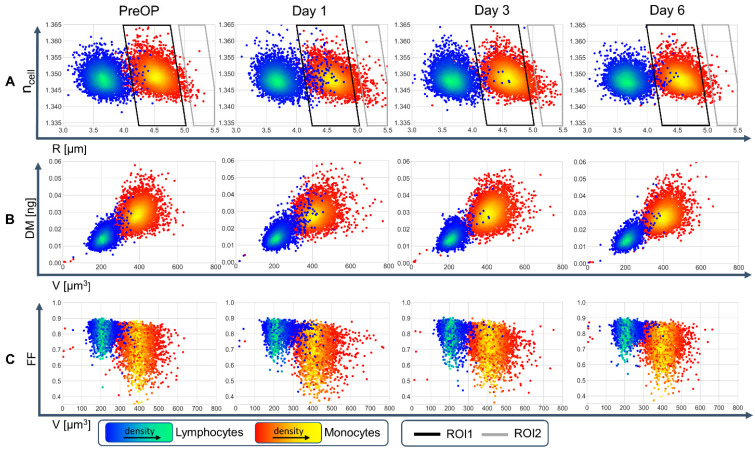
Density scatterplots of biophysical cell parameters during the perioperative course. An animated graphical representation for dynamical visualization is provided in Supplement File S2 file. (**A**) Initially determined parameters refractive index *n*_cell_ vs. cell radius R, and subsequently calculated distributions of (**B**): dry mass DM vs. volume *V*, (**C**): cell shape related form factor FF vs. V. Data were obtained from 150 lymphocytes and 150 monocytes from blood samples of all 25 patients acquired preoperatively (PreOP) as well as postoperative on day 1, 3 and 6. The two leucocyte fractions appear clearly separated in all scatterplots. Preoperatively, only few cells scatter out of the main population of lymphocytes and monocytes. Scattering of cells with enlarged volume increases postoperatively at day 1 in all density plots (**A**–**C**) and causes an increased overlap between the data clouds of the different cell types (ROI1 in A: lymphocytes, ROI2 in A: monocytes, an animation is provided in Supplement S2 file). Consecutively, the overlap decreases again towards the initial distribution at PreOP. Data in each column correspond to biophysical parameters retrieved from the same cell populations. A corresponding density scattering plot *n*_cell_ vs. *V* for all patients as well as scatterplots for all individual patients are provided in Figure S2 of Supplement S1 file and Supplement S3 file.

The resulting density scatterplots from a single patient (Figure 2D1–D3) show different combinations of biophysical cell parameters (*n*_cell_ vs. R, DM vs. V and FF vs. V) and allow a clear differentiation between monocytes and lymphocytes. This differentiation of monocytes and lymphocytes is also observable in the entire data set from all 25 patients (Figure 4), revealing clearly defined populations in which only small fractions of cells scatter out of the main population at PreOP. Scattering of cells with enlarged radius and corresponding volume is observed at day 1 after operation (see region of interests (lymphocytes: ROI1, monocytes: ROI2) indicated by parallelograms in Figure 4A, and Supplementary Animation S2) and decreased on days 3 and 6, again towards the initially measured distribution at PreOP.

### 3.3. Cell Volume, Refractive Index and Form Factor Change Significantly during Perioperative Course

**Figure 5:**
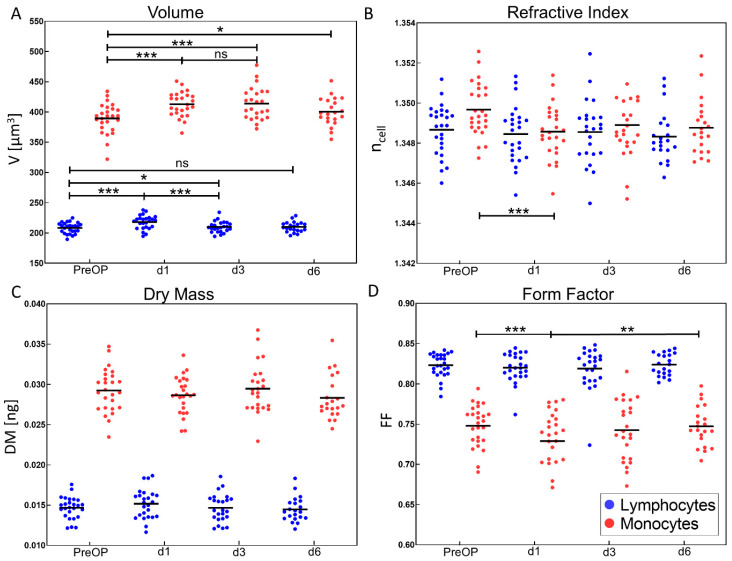
Biophysical DHM parameters of monocytes and lymphocytes during perioperative course. Each data point represents the average value from a fraction of 150 cells that were analyzed per time point and individual patient. The black horizontal line indicates the respective mean value of all patients per time. Parameters were determined immediately before cardiac surgery (PreOP) as well as postoperatively at day 1 (d1), 3 (d2) and 6 (d6). In analogy to the scatter plots in Figure 4, monocytes and lymphocytes are clearly differentiated regarding volume V (**A**), dry mass DM (**C**) and form factor FF (**D**), which was used to quantify the deviation of the cell shape from a sphere. While average monocyte volume and dry mass are higher than lymphocyte volume, form factor values of monocytes are in general lower than lymphocyte values. Regarding the refractive index *n*_cell_ (**B**), no significant differences between the two cell populations are detected. * *p* < 0.05; ** *p* < 0.01, *** *p* < 0.001, ns = not significant; a graphical representation of the corresponding standard deviations for all biophysical parameters in (**A**–**D**) is provided in Figure S3 in Supplement S1 file. Numerical mean values ± SD and SEM for all mean values of all patients are listed in Table S4 in Supplement S1 file. Figure S4 in Supplement S1 file illustrates corresponding perioperative lymphocyte volume trends of individual patients for three subgroups based on CRP level, epinephrine treatment, and complicated postoperative course as identified in Figure 6.

Figure 5 shows perioperative scatterplots of the average values for cell volume, dry mass, refractive index and form factor, obtained from 150 lymphocytes and 150 monocytes for each patient as illustrated in Figure 2D1–D3. Lymphocytes and monocytes can be clearly distinguished for volume (Figure 5A), dry mass (Figure 5C), and form factor (Figure 5D) data, but not by the obtained highly similar refractive index values (Figure 5B).

The average lymphocyte volume of all patients (black horizontal lines in Figure 5A) increased significantly after surgery from 208 ± 9 µm^3^ to 218 ± 11 µm^3^ at d1 compared to PreOP values while the volume of corresponding monocytes increased from 390 ± 25 µm^3^ to 413 ± 20 µm^3^. From d1 to d3 no significant change of monocyte volume (413 ± 20 µm^3^ and 414 ± 27 µm^3^) was detected whereas a significant difference between d3 (414 ± 27 µm^3^) and PreOP (390 ± 25 µm^3^) was observed. Lymphocyte mean volume decreased significantly from 218 ± 11 µm^3^ on d1 to 210 ± 9 µm^3^ on d3 and showed a less significant difference between PreOP and d3. While the mean lymphocytes volume on d6 (210 ± 9 µm^3^) almost decreased to PreOP values (208 ± 9 µm^3^), monocyte mean volume between d6 (400 ± 23 µm^3^) and PreOP (390 ± 25 µm^3^) was still found significantly increased. The observed perioperative course of the average volume values was in line with shifts of the corresponding entire cloud of the individual patient data in Figure 5A.

Averaged refractive indices, quantifying the concentration of the intracellular solutes of monocytes and lymphocytes, for all patients ranged from 1.3454 ± 1 × 10^−4^ to 1.3524 ± 1 × 10^−4^ with a strong overlap of the data (Figure 5B). The average refractive index of monocytes showed a significant decrease between PreOP (1.3497 ± 1 × 10^−4^) and d1 (1.3485 ± 1 × 10^−4^). For lymphocytes a similar trend was observed, but this was not significant.

The dry mass (DM) course of both monocytes and lymphocytes remained constant without any statistically significant change during the entire observation period (Figure 5C).

Form factor (FF) of monocytes as an indicator for changes of cell shape significantly decreased on d1 from 0.75 ± 0.03 to 0.73 ± 0.04 and increased again on the following days until d6 to the initial PreOP values (Figure 5D). In contrast, for lymphocytes no statistically significant FF changes were observed.

In addition to the average biophysical cell parameters of individual patients as presented in Figure 5, the corresponding standard deviations were also analyzed (see graphical representation in Figure S3 in Supplement S1 file). For both, lymphocyte and monocyte average volume, on d1 a significantly increased standard deviation (lymphocytes: *p* < 0.001, monocytes: *p* < 0.001) was observed compared with PreOP which decreased towards the initial distributions during d3 and d6 (Figure S3A). These findings correspond with the scattering of the cell radius values observed in the ROIs for individual cells in Figure 4 at day 1.

### 3.4. Synchronous Changes in Biophysical DHM Data, Flow Cytometric Markers, Routine Laboratory Parameters, and Drug Dosages Revealed by Bivariate Correlation

The DHM data in Figure 4 and Figure 5 indicate significant changes in the biophysical parameters at day 1 after surgery. With the aim of finding crosslinks to clinically relevant parameters, DHM physical parameter changes between day 1 and the day prior to surgery (d1-PreOP) in Figure 5 were bivariately correlated with the respective differences in flow cytometric markers, retrieved as described in Section 2.5 and routine laboratory data. Table 1 lists the obtained parameter correlations for d1-PreOP in descending order of Pearson correlation coefficient and statistical significance. Two heatmaps presenting *p* values and correlation coefficients of all parameter changes for d1- PreOP are provided in Figures S7 and S8 in Supplement S4 file.

Prominent changes in biophysical DHM parameters correlated with flow cytometric surface marker CD19^+^ (B-cells), T cell activation marker CD86^+^ (B cells and monocytes), monocytic CD206^+^ cells, and antigen presenting complex monocytic HLA-DR (mHLA-DR). Additionally, correlations between monocyte volume changes and percentage alterations of necrotic/late apoptotic cells as well as between lymphocyte volume changes and epinephrine dose were detected.

### 3.5. DHM Parameter Changes Correlated Significantly with Complicated Course, Epinephrine Treatment and Inflammation Marker CRP

**Figure 6:**
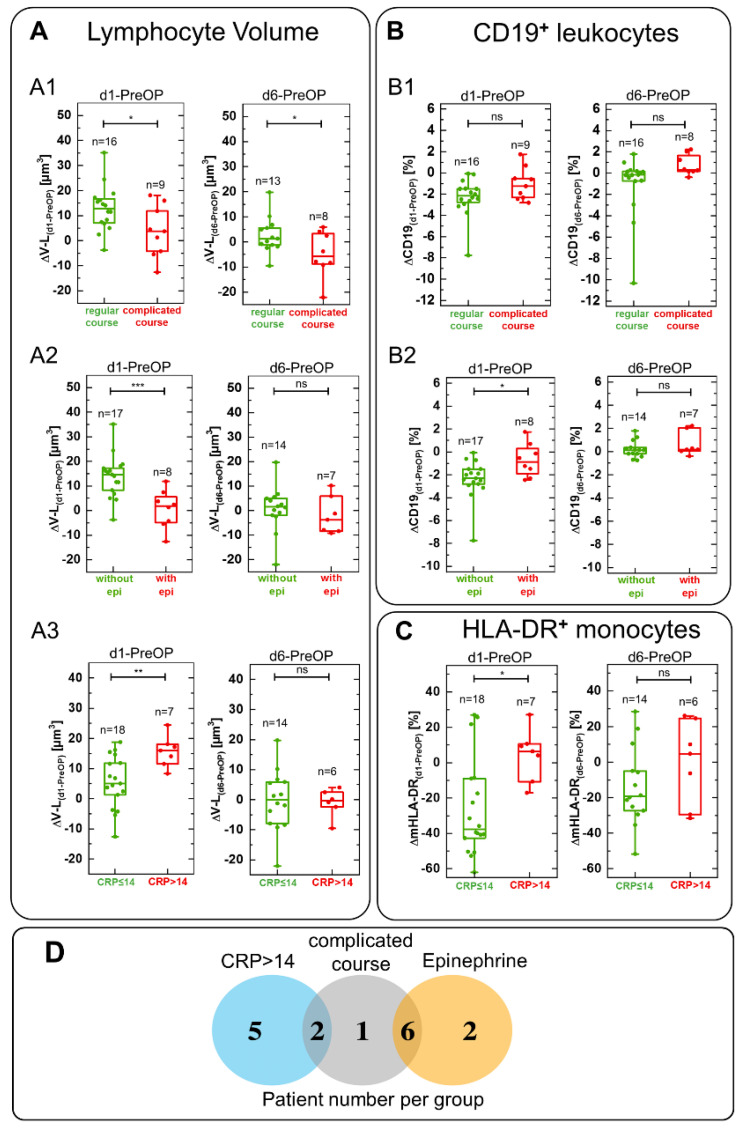
Lymphocyte volume changes ΔV-L_(d1-PreOP)_, ΔV-L_(d6-PreOP)_ at day 1 vs. the day prior surgery (PreOP) and at day 6 after surgery vs. PreOP detected by DHM compared with accompanied changes in flow cytometric markers and laboratory parameters. (**A1**) patients divided into groups based on regular and complicated postoperative course. (**A2**) patients divided into groups based on treatment with and without epinephrine. (**A3**) Patients divided into groups concerning different CRP levels (CRP≤ or >14 mg/dL). Lymphocyte cell volume changed significantly between all three defined groups on d1-PreOP (**A1**–**A3**). Patients with a complicated postoperative course and those who received epinephrine showed no change in lymphocyte volume (**A1**,**A2**) while a CRP level >14 mg/dL correlated with a significant increase in lymphocyte volume (**A3**). Differences in relative number of B cells among leukocytes (**B1**, CD19^+^_(d1-PreOP)_, and CD19^+^_(d6-PreOP)_) were not significant but showed an increasing trend in case of a complicated postoperative course. In the epinephrine treatment group ΔCD19^+^_(d1-PreOP)_ increased significantly (**B2**) while ΔCD19^+^_(d6-PreOP)_ was not significantly altered (**B2**). Difference of monocyte ΔmHLA-DR_(d1-PreOP)_ decreased significantly in patients with CRP ≤ 14 mg/dL compared to those with CRP >14 mg/dL and was insignificant at d6-PreOP (**C**). Venn diagramm of patients groups with CRP > 14 mg/dL, complicated postoperative course, or treated with epinephrine (**D**). No patient with epinephrine administration showed high CRP levels (>14 mg/dL, **D**). Δ indicates parameter changes between different measurement days after surgery and the day prior surgery: d1-PreOp and d6-PreOP. * *p* < 0.05; ** *p* < 0.01, *** *p* < 0.001, ns = not significant.

With the aim of exploring if biophysical DHM parameters are suitable to identify patient clusters with increased inflammatory response, patients were dichotomized in groups concerning course (regular or complicated), severity of inflammation as indicated by CRP levels (CRP > 14 mg/dL or CRP ≤ 14 mg/dL), and treatment with or without epinephrine. Figure 6A1–A3 show the obtained results as box blot representation and the corresponding Venn diagram (Figure 6D). Out of the group of nine patients with severe course, six individuals were treated with epinephrine while two also showed a CRP level > 14 mg/dL. No overlap was found between the group of epinephrine treated patients and the group with CRP > 14 mg/dL. 

Lymphocyte volume difference ΔV-L_(d1-PreOP)_ was found to be significantly increased in patients with a regular course or not treated with epinephrine. On the other hand, patients with a complicated course and treated with epinephrine showed almost no change in lymphocyte volume difference ΔV-L_(d1-PreOP)_ (Figure 6A1,A2). In contrast, lymphocyte volume difference increased in patients with severe inflammation as indicated by CRP >14 mg/dL (Figure 6A3). All volume changes were no longer significant at day 6 (Figure 6A1–A3), except of the patient group with complicated courses (Figure 6A1). The results in Figure 6A1–A3 agree with the perioperative trends of the absolute lymphocyte volume for the main fraction of individual patient courses of the three groups (Figure S4 in Supplement S1 file). Changes in the relative amount of B cells (ΔCD19_(d1-PreOP)_) indicated a decreasing trend in patients with regular course but were not significant (Figure 6B1), and the same was the case for ΔCD19_(d6-PreOP)_ (Figure 6B1). ΔCD19_(d1-PreOP)_ level changes were significantly decreased compared to patients treated with epinephrine (Figure 6B2) and normalized again at day 6 (ΔCD19_(d6-PreOP)_, Figure 6B2). CRP value did not correlate with ΔCD19 (data not shown). The difference ΔmHLA-DR _(d1-PreOP)_ in the inflammation relevant laboratory parameter mHLA-DR correlated with CRP blood concentration (Figure 6C) and correlation decreased to a non-significant level at day 6 (ΔmHLA-DR _(d1-PreOP)_, Figure 6C).

**Figure 7:**
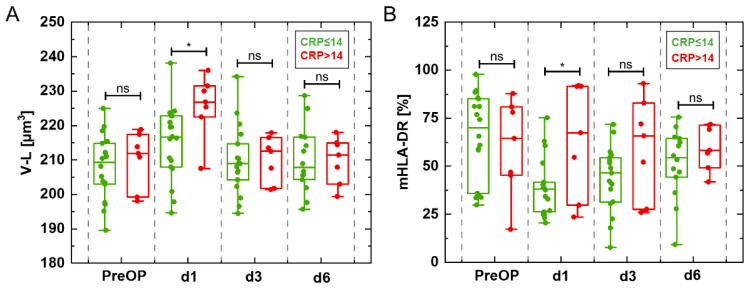
Comparison of changes in (**A**) Lymphocyte volume V-L and (**B**) mHLA-DR at single measurement days (PreOP, d1, d3, d6) in patient groups dichotomized based on CRP values (CRP ≤ 14 mg/dL, CRP > 14 mg/dL). Postoperatively, at day 1 (d1) lymphocyte volume increased synchronous with an enlarged mHLA-DR level. * *p* < 0.05, ns = not significant.

Moreover, for patients with regular postoperative course or complicated course, (CRP > 14 mg/dL or CRP ≤ 14 mg/dL) and treatment with or without epinephrine, cell volume, refractive index and dry mass were compared with inflammation flow cytometric cell surface markers at single measurement days during the perioperative course. We observed a significant increase in lymphocyte volume V-L and inflammation relevant antigen presenting complex mHLA-DR level at day 1 in patients with a CRP level > 14 mg/dL compared to those with CRP ≤ 14 mg/dL (Figure 7A,B). For all other groups V-L and mHLA-DR did not significantly change (data not shown) although changes in platelets, T cells (CD3^+^), T helper cells (CD4^+^), and apoptotic cells were present (Figure S5 in Supplement S1 file).

## 4. Discussion

The aim of this pilot study was to investigate the relevance of biophysical blood cell parameters to describe and follow up inflammatory processes and the clinical course of patients undergoing cardiac surgery with cardiopulmonary bypass. We manually measured about 30,000 monocytes and lymphocytes isolated from blood samples taken perioperatively from 25 patients on different days and compared the obtained biophysical information to flow cytometric surface marker data and routine laboratory parameters. Clinically, our study is motivated by previous research which indicates that the intraoperative use of CPB leads to a systemic inflammatory response that is induced by contact activation of blood due to the artificial surface of an extracorporeal system [47,48,49]. Although intraoperative complications in elective cardiac surgery are rare due to high operative standards [50], there is a 40% chance of developing post-surgery complications such as bleeding, pneumonia or acute kidney injury [51,52,53,54]. This generates the demand for sophisticated methods of detection for inflammatory processes in blood that are more closely associated with postoperative complications. The current gold standard to investigate inflammation related blood cell alterations is marker-based FCM which is highly specific but also bears several challenges due the involved complex multiparametric measuring and laborious staining protocols. We thus explored in our study quantitative phase imaging (QPI) [18] for PBMC analysis which provides access to absolute physical cell parameters and simplified sample preparation without labeling. In detail, we compared biophysical cell data acquired by QPI based on DHM with selected and acknowledged inflammation related flow cytometric blood cell markers and routine laboratory data. DHM is a minimally invasive quantitative optical microscopy technique which allows investigations of living cells [19]. Moreover, it allows the precise calculation of the absolute volume [28], dry mass [42], detection of water efflux/influx [55], early cell death [56] and cell shape [57]—all parameters with possible inflammation relevance.

Blood monocytes and lymphocytes were isolated label-free, to modify cells as little as possible in the preparation process for DHM investigations and were separately analyzed for changes in volume, refractive index, dry mass and form factor. To analyze the reproducibility of DHM parameter retrieval, a bootstrap analysis was employed as described in Section 2.6. The resulting Benjamini-Hochberg corrected sample *p* values are indicative of a high stability of the results within an SD corridor of ± 0.3, and support the observed highly significant parameter changes during the perioperative course. Figure 2D1–D3 and Figure 4 show that lymphocytes and monocytes can be clearly distinguished by their biophysical properties except for the cellular refractive index. Especially, the cell volume (mean for all 15,000 measured monocytes: 404 ± 25 µm^3^; mean for all measured 15,000 lymphocytes: 212 ± 10 µm^3^, Figure 4 and Figure 5) allowed a particular differentiation. The clear identification of the different cell types for data from individual patients (Figure 2D1–D3 and Supplement S3 file) suggests that the complex cell isolation procedures as applied in this pilot study may be not necessary in the future prior to DHM analysis by developing simplified protocols. 

In Figure 4 and the animation in Supplement S2 file, it is observed that some of the cells scatter out of their main population especially at day 1 after operation, while scattering abates over day 3 to day 6. However, due to the variability of the investigated primary cells and the limited number of 150 manually analyzed cells per cell type in our pilot study, no clear trends, as visible in the clouds for the averaged values of the entire patient group in Figure 5, were observed during the perioperative courses of individual patients. The cell scattering effects observed in Figure 4 could be explained by subgroups of monocytes and lymphocytes with noticeable changes due to inflammatory processes [35] and are in agreement with significant changes in leucocyte count, size and granularity that has been reported in the literature for measurements of monocyte distribution in immediate postoperative processes [10,11]. The observed scattering effects also conform with earlier findings on volume and scatter changes that were identified as possible parameters for the early detection of a severe systemic inflammation up to septic shock [9,10,58,59,60].

In Figure 5 the mean values of lymphocyte and monocyte volume for each patient were plotted to visualize changes of monocyte and lymphocyte volume and changes during the perioperative course. Volume changed significantly post-operation, especially at day 1 in both populations (Figure 5A), while showing a tendency to a reduced refractive index in lymphocytes while monocyte refractive index decrease was significant (Figure 5B). Dry mass was constant over the entire observed period (Figure 5C). These correlations suggest an uptake of water responsible for volume increase [60]. The enlarged standard deviation obtained for the average lymphocyte and monocyte volume at d1 compared with PreOP, d3 and d6 (Figure S3A in Supplement S1 file) corresponds with the increased scattering of the single cell radius values observed for individual cells at day 1 (see ROIs in Figure 4). Changes in form factor FF (Figure 5D) as an indicator for changes of the cell shape (here change with respect to a spherical appearance) were significant (*p* < 0.001) for monocytes but not lymphocytes The observed cell shape changes are small (∆*FF* ≈ −0.02) but indicate less spherical cells, which might be explained by changes in surface marker expression and conformational alterations.

Moreover, the lymphocyte volume development found for the entire patient collective (black horizontal lines in Figure 5) was also reflected by the majority of the individual patient courses (Figure S4 in Supplement S1 file) for the subgroups identified in Figure 6D (CRP level, epinephrine treatment, and complicated postoperative course). However, no such clear trends were detected for all other biophyiscal parameters in Figure 5.

Bivariate correlation of all determined parameter (see heatmaps in Figures S7 and S8 in Supplement S4 file) revealed statistical significances (*p* values from 0.05 to 0.01) between DHM parameter changes and alterations of flow cytometric markers and epinephrine dose prior- and post-surgery (d1-PreOP) (Table 1). The corresponding absolutes of the resulting correlation coefficients ranged from 0.401 to 0.514 and reflect the patient dependent variability of the investigated primary cells (Figure 4 and Figure 5) (see representative correlation plot for ∆V-L vs. ∆CD19_abs_ in Figure S6 of Supplement S1 file). In particular, for the average of the entire patient collective we found a negative correlation of lymphocyte volume decrease ΔV-L_(d1-PreOP)_ with an increase ΔCD19_(d1-PreOP)_ of CD19 positive B cells. CD19 positive B cells play an important role in the inflammatory response by promoting T cell response and therapy related site effects [12,13]. Refractive index changes Δ**n*_cell_*-L_(d1-PreOP)_ of lymphocytes were positively correlated with changed numbers of CD86-expressing B cells (ΔCD86_(d1-PreOP)_). A high CD86 expression on lymphocytes is accompanied with T cell activation and with an increase of proinflammatory cytokines [61]. A similar correlation was found for difference in monocytic refractive index Δ**n*_cell_*-M_(d1-PreOP)_ and changes in monocytic differentiation markers ΔCD206_(d1-PreOP)_, both CD86 and CD206 expression on monocytes. These markers are key players in inflammation promoting T-cell response. This indicates that the refractive index change Δ**n*_cell_*-M_(d1-PreOP)_ is associated with cell differentiation processes, as observed earlier [62]. Furthermore, the increased differences in form factor of monocytes Δ*FF*-M_(d1-PreOP)_ correlate significantly (*p* < 0.05) with increased changes in ΔmHLA-DR_(d1-PreOP)_ expression. Decreased mHLA-DR correlates with loss of activity of monocytes during inflammatory course and in general with a higher mortality after septic shock [13]. Changes in surface protein conformation and expression may explain the observed form factor changes. In addition, both, refractive index (Δ*n*_cell_-M_(d1-PreOP)_) and volume increase (Δ*V*-M_(d1-PreOP)_) correlated positively with increased numbers of apoptotic and necrotic PBMCs (Δlate apoptotic/necrotic cells), which may be explained by cell death induced cell swelling [56,63].

To validate DHM parameters as tools for description of clinical outcomes and inflammatory processes, we identified patient subgroups (see Figure 6D). The first subgroup consisted of nine patients suffering from a postoperative complicated course (e.g., development of acute kidney injury or pneumonia) after major cardiac surgery with cardiopulmonary bypass (Table S1 in Supplement S1 file). The second group of eight patients was chosen based on administration of epinephrine due to intra- and postoperative circulatory stabilization, while the third subgroup included seven patients with profound postoperative increase in CRP (>14 mg/dL) [46]. 

The first subgroup of patients showed no difference in lymphocyte cell volume ΔV-L_(d1-PreOP)_ compared to patients with regular course, in which a significant increase was found (Figure 6A1), and agrees with the courses of the trends of the absolute cell volume for individual patients (Figure S4A in Supplement S1 file). The observed difference may be explained by an immune paralysis of circulating lymphocytes after cardiopulmonary bypass, which might increase the susceptibility to develop postoperative complication [64,65]. This is also in agreement with the relative numbers of T cells (CD3^+^) and T helper cells (CD4^+^), as effector cells of the adaptive immune system, which were significantly lower in the group with complicated courses at day 1, day 3 and day 6 (Tables S2 and S3 in Supplement S1 file).

In the second subgroup patients were divided based on administration of epinephrine. Epinephrine is an agonist binding to alpha- and beta-adrenergic receptors with effects on the cardiovascular system [66], endocrine system and immune system [67]. In addition, epinephrine promotes the aggregation of platelets [68] and may contribute to the low platelet concentrations in epinephrine treated patients (Figure S5D in Supplement S1 file), which are known to be associated which postoperative bleeding [69] and increased mortality. In patients who did not receive epinephrine for cardiocirculatory support, the difference in lymphocyte cell volume ΔV-L_(d1-PreOP)_ was significantly increased on the first postoperative day; in contrast to the patients treated with epinephrine, in whom the difference in lymphocyte cell volume ΔV-L_(d1-PreOP)_ did not change at the first postoperative day (Figure 6A2), accompanied by an increase in relative B cells (CD19^+^ cells) counts (Figure 6B2). Both parameters normalized at the sixth postoperative day (Figure 6A2,B2). These courses correspond to the temporal development of the absolute cell volume of the individual patients Figure S4B in Supplement S1. Furthermore, these findings correlate with the observation that epinephrine can selectively reduce numbers of circulating immune cells, for instance NK cells and CD8^+^ T cells in patients with heart failure [67,70,71]. These cells differ in their biophysical parameters from B cells for example in a slightly larger cell volume and are distinguishable by QPI analysis [35]. Therefore, a potential loss of circulating cells with increased volume may explain the unchanged average cell volume in epinephrine treated patients. Our findings along with the observed volume changes and increase in scattering day 1 (see ovals in Figure 4 and Figure S2 in Supplement S1 file) support the thesis that, at least in the case of lymphocytes, observed alterations could be caused by changes in circulating leukocyte subpopulations, which could not be separated in our study in the DHM analysis. 

The third group consisted of patients with CRP levels > 14 mg/dL at the first postoperative day. CRP is a surrogate of cardiac surgery associated inflammation. It is known that CRP plasma levels can increase by over 50 mg/dL within the first days of severe tissue damage such as that caused by surgery [72]. A main inducer of CRP gene expression is IL-6 [73]. Monocytes release IL-6 after direct or indirect activation by damage-associated molecular patterns (DAMPs), for instance from damaged or dying cells after trauma or surgery [74]. It is known that coronary artery bypass grafting is associated with immunoparalysis of monocytes and reduced release of IL-6 [65]. IL-6 together with mHLA-DR, can be used to quantify cardiac surgery associated immune suppression [65]. This might explain the observation that patients in the group with lower CRP concentrations postoperatively at day 1 had relatively more inactive monocytes as indicated by a significant decrease of ΔHLA-DR_(d1-PreOP)_ (Figure 6C). In contrast, in patients with high CRP we found a significant increase in lymphocyte cell volume difference ΔV-L_(d1-PreOP)_ (Figure 6A3) which matches the major fraction of tracks for the absolute cell volume found for individual patients (Figure S4C in Supplement S1 file). Both, ΔHLA-DR_(d6-PreOP)_ and lymphocyte cell volume ΔV-L_(d6-PreOP)_ normalized at day 6 (Figure 6A3,C). In-line with these observations, a significant increase was found at day one after surgery for the absolute volume and mHLA-DR for CRP levels > 14 mg/dL compared to CRP ≤ 14 mg/dL (Figure 7A,B). This correlation might be explained by finding of previous studies, for instance reported by Albertsmeier et al. [12], that T cells and activated monocytes mutually influence each other. T cells may be upregulated or downregulated by activated monocytes and the release of cytokines from monocytes can be induced and triggered by T cells in turn [12].

The Venn diagram in Figure 6D illustrates that the group of patients receiving epinephrine for circulatory support and the group of patients that developed a complicated postoperative course show a noticable overlap. This may explain the similar trends of the DHM parameters in both groups. In contrast, no overlap of patients with elevated CRP (>14 mg/dL) and epinephrine treatment is observed, which corresponds to an immunosuppressive effect of epinephrine by beta-adrenergic receptor stimulation and consecutively lower CRP expression, as earlier reported [72,75].

In sum, the results in Figure 4, Figure 5, Figure 6 and Figure 7 suggest several crosslinks between biophysical parameters accessible by QPI with DHM and inflammation related cytometric markers, as well as laboratory and clinical parameters.

A current technical bottleneck in our approach is the time consumption for the experimental data acquisition. This involved the manual selection and numerical evaluation of DHM QPI images of single cells in suspension and took, in this pilot study, about 2 h for the measurement of 150 cells per cell type at a single time point for each individual patient. However, the combination of our method with microfluidics approaches and a sophisticated evaluation of QPI images [40,76], hydrodynamic focusing of the samples in a laminar flow stream at velocities suitable for imaging flow cytometry [77,78], and rapid camera hardware promise increased automation and significant decrease of hologram acquisition times down to the millisecond range. Further acceleration can be expected from advanced image processing strategies utilizing sophisticated numerical procedures [79], and integration of fast graphics processing units (GPUs) [80], with prospects to speed up DHM QPI image reconstruction beyond video frequency (e.g., >25 Hz). 

As already mentioned in the discussion of the results in Figure 4, cells that were activated pro- or anti-inflammatory presumably leaded an increased heterogeneity of biophysical cell parameters. Here, analysis of the data available by QPI with sophisticated evaluation concepts, e.g., based on machine learning algorithms that allow considering of multiple parameters [35,76,81,82], promises further insights into our data sets and into the identification of additional cell subfractions.

## 5. Conclusions and Future Perspectives

In summary, the results of the statistical evaluations in our study suggest several crosslinks between biophysical parameters of DHM analysis and inflammation related flow cytometric, as well as laboratory and clinical parameters. This was the first time that DHM was used in a larger study on 25 cardiac surgery patients, in which 30,000 cells were measured and analyzed. In particular, our data shows that DHM allows a clear differentiation of lymphocytes and monocytes based on the calculation of refractive index, volume, dry mass and cell shape related form factor and, despite the limited number of patients of this pilot study, also provided promising correlations with state-of-the-art flow cytometry markers, epinephrine treatment and CRP level changes. The data from this study pave the way for future in-depth studies on the underlying mechanisms of the observed changes in biophysical cell parameters, and to recover associations with further inflammation related markers and cell types, as well as for prospective usage as a diagnostic.

## Figures and Tables

**Table 1:** Bivariate correlation of DHM biophysical parameter changes with alterations of flow cytometric markers and epinephrine dose prior to surgery and at day 1 post-surgery (d1-PreOP). Δ indicates parameter changes between different measurement days after surgery and the day prior to surgery: d1-PreOP. Correlations are listed in a descending order according to Pearson correlation coefficient and significance. Only significant correlations are listed. * *p* < 0.05; ** *p* < 0.01.

**Correlations between DHM parameter changes and alterations of flow cytometric markers and epinephrine dose prior- and post-surgery (d1-PreOP)** Δ indicates parameter differences between measurement days d1 and PreOP (d1-PreOP)		
**Parameter 1**	**Parameter 2**	**Pearson** **Correlation Coefficient**
∆V-L	∆CD19_abs_	−0.514 **
∆V-L	∆Epinephrine dose	−0.484 *
∆V-M	∆Necrosis/late ∆apoptosis	0.479 *
∆*n*_cell_-L	∆CD86	0.464 *
∆FF-M	∆mHLA-DR	0.464 *
∆*n*_cell_-M	∆Necrosis/late apoptosis	−0.44 *
∆V-M	∆*n*_cell_-M	−0.431 *
∆*n*_cell_-M	∆mCD206	0.405 *
∆FF-M	∆*n*_cell_-M	0.401 *

Abbreviations: V-L: Lymphocyte volume; FF-M: Monocyte form factor; V-M: Monocyte volume; *n*_cell_-L: Lymphocyte refractive index; *n*_cell_-M: Monocyte refractive index; Flow cytometric markers: CD19_abs_; Necrosis/late apoptosis; CD86; monocytic HLA-DR (mHLA-DR); monocytic CD206 (mCD206). M, m: monocyte related parameters and L: lymphocyte related parameters.

## Data Availability

Data is available within the paper and in the Supplementary Materials.

## Supplementary Materials

The following supporting information can be downloaded at: https://www.mdpi.com/article/10.3390/cells11040755/s1, Supplement S1 file: Supplementary Table S1: Patient’s data corresponding to measurements and analysis in Section 2. Supplementary Table S2: Flow cytometric marker analysis corresponding to Figure 1. Supplementary Table S3: Materials, associated dyes and manufacturer used for flow cytometric marker analysis corresponding to Figure 4. Supplementary Table S4: Mean values and standard deviation of the mean data as well as standard error for all patients. Supplementary Figure S1: Bright-field images of monocytes and lymphocytes. Supplementary Figure S2: Density scatterplots corresponding to Figure 4. Supplementary Figure S3: Standard deviations of the average values in Figure 5. Supplementary Figure S4: Individual courses of lymphocyte volume for three patient subgroups corresponding to Figure 6D. Supplementary Figure S5: Further comparisons of parameters between the three subgroups corresponding to Figure 6D. Supplementary Figure S6: Representative correlation plot corresponding to Table 1; Supplement S2 file: Animation corresponding to Figure 4; Supplement S3 file: Density scatterplots of all individual patients (n = 25); Supplement S4 file: Supplementary Figures S7–S8. *p* values and correlation coefficient of all data presented in heat maps. References [83,84,85,86,87,88,89,90] are citied in the Supplementat Materials.
