# Supplementary material for: Digital Holographic Microscopy for Label-Free Detection of Leukocyte Alternations Associated with Perioperative Inflammation after Cardiac Surgery
Source: Cells. 2022 Feb 21;11(4):755. doi: 10.3390/cells11040755 (PMC8869820; doi:10.3390/cells11040755)

Digital holographic microscopy for label-free detection of leukocyte alternations associated with perioperative inflammation after cardiac surgery

Steike DR[1†], Hessler M[2†], Korsching E[3], Lehmann F[2], Schmidt C[2], Ertmer C[2], Schnekenburger J[4], Eich HT[1], Kemper B[4*], Greve B[1*]

[1] Department of Radiotherapy -Radiooncology-, University Hospital Muenster, Albert-Schweitzer-Campus 1, Building A1, 48149 Muenster, Germany

[2] Department of Anesthesiology and Intensive Care and Pain Medicine, University Hospital of Muenster, Albert-Schweitzer-Campus 1, 48149 Muenster, Germany

[3] Institute of Bioinformatics, Medical Faculty, University of Muenster, 48149 Muenster, Germany

[4] Biomedical Technology Center, Medical Faculty, University of Muenster, 48149 Muenster, Germany

*** Corresponding authors:**

Dr. Björn Kemper
Biomedical Technology Center, University of Muenster
Mendelstraße 17
D-48149 Muenster
Email: bkemper@uni-muenster.de

Prof. Dr. Burkhard Greve
Department of Radiotherapy -Radiooncology-, University Hospital Muenster, Albert-Schweitzer-Campus 1, Building A1
D-48149 Muenster, Germany
Email: greveb@uni-muenster.de

Table S1. Patient's data corresponding to measurements and analysis in section *2 Materials and Methods*

**Table S1 Baseline patient characteristics** (unit)		
**N**		25
**Sex**	male	16
	female	09
**Age** (years)		67.1±15.3
**Body weight** (kg)		78±22
**Routine laboratory parameter**		
Creatinine in plasma (mg/dL)		1.1±0.4
Creatinine clearance (mL/min/1.73·m^2)		67.6±22.6
CRP (mg/dL)		1.0±1.0
Hemoglobin concentration (g/dL)		13.4±2.2
Leukocytes (x10^9/L)		7.5±1.6
Platelets (x10^9/L)		208±42
**Surgical procedure**		
CABG		12
Aortic valve replacement		7
Mitral valve reconstruction		5
Tricuspid valve reconstruction		3
Combination CABG and valve surgery		4
Dual valve surgery		2
**Number of severe complications**		8 (36%)
**Patients with more than one complication**		7 (28%)
Acute kidney injury		1
Cardiopulmonary resuscitation		3
Heart failure, cardiogenic shock, or myocardial infarction		5
Pneumonia or respiratory failure		3
Neurological complications (delirium or stroke)		3
Need for surgical revision		3

Legend: If appropriate, data were presented as mean with standard deviation.

Abbreviations: *CABG*, coronary artery bypass grafting; *CRP*, c reactive protein.

Table S2. Flow cytometric marker analysis corresponding to Figure 1. Flow cytometry was used to identify and quantify leucocyte subsets by their surface marker expression, which were described to be relevant for inflammatory processes

Surface marker	Cell type/function	Literature
**CD45**	Leukocytes	[1]
**CD3**	T cells	[1–3]
**CD4**	T helper cells	[1–3]
**CD19**	B cells	[1]
**CD39**	Regulatory T cells	[1]
**mCD16**	Monocyte subpopulations	[1,4–6]
**CD86$^+$ B cells**	Costimulatory signals for T cell activation	[1,7]
**CD86$^+$ monocytes**	Costimulatory signals for T cell activation	[1,7]
**CD14**	Monocytes	[1,8]
**CD206**	Adaptive immunity	[1,9]
**TNFR1**	Programmed cell death	[1]
**mHLA-DR**	T cell receptor ligand, important for sepsis prognosis	[1,8,10]
**mPD-L1**	Involved in suppression of the adaptive immune system	[1,11]

Table S3. Materials, associated dyes and manufacturer used for flow cytometric marker analysis corresponding to Figure 4

**Antibody**	**Fluorochrome**	**Manufacturer**
CD45	FITC	Becton Dickinson
CD3	APC	Becton Dickinson
CD4	APC	Immunotools
CD19	APC	Becton Dickinson
CD39	PE	Miltenyi
CD16	PE	Miltenyi
CD86	FITC	Miltenyi
CD14	VioBlue	Miltenyi
CD206	APC	Becton Dickinson
TNFR1	PE	BioCarta
mHLA-DR	PE	Immunotools
PD-L1	BB515	Becton Dickinson
Isotype	FITC	Becton Dickinson
Isotype	PE	Miltenyi, BioCarta, Immunotools

Table S4. Mean values and standard deviation of the mean data as well as standard error for all patients (n=25) each measured day for both cell types (monocytes and lymphocytes) corresponding to Fig. 5; 5 biophysical parameters; descriptive statistics calculated with GraphPad Prism 9.1, GraphPad Software, San Diego, USA

	Lymphocytes				Monocytes			
**Time point**	**PreOP**	**Day 1**	**Day 3**	**Day 6**	**PreOP**	**Day 1**	**Day 3**	**Day 6**
**Volume** (µm³)								
Mean ± standard deviation	208.3 ± 8.8	218.0 ± 11.2	209.9 ± 9.1	210.0 ± 8.6	389.5 ± 24.7	412.7 ± 20.4	414.0 ± 26.5	400.4 ± 23.1
standard error	1.7	2.2	1.9	1.9	4.8	4.1	5.4	5.0
**Radius** (µm)								
Mean ± standard deviation	3.67 ± 0.05	3.72 ± 0.06	3.68 ± 0.05	3.68 ± 0.05	4.52 ± 0.10	4.61 ± 0.08	4.61 ± 0.10	4.56 ± 0.09
standard error	0,01	0.01	0.01	0.01	0.02	0.02	0.02	0.02
**Dry Mass** (ng)								
Mean ± standard deviation	0.0147± 0.0014	0.0152± 0.0018	0.0147± 0.0017	0.0145± 0.0015	0.0293± 0.0027	0.0287± 0.0023	0.0295± 0.0031	0.0283± 0.0027
standard error	0.0003	0.0004	0.0004	0.0003	0.0005	0.0005	0.0006	0.0006
**Refractive Index**								
Mean ± standard deviation	1.349 ± 0.001	1.348 ± 0.001	1.349 ± 0.002	1.348 ± 0.001	1.350 ± 0.001	1.349 ± 0.001	1.349 ± 0.002	1.349 ± 0.001
standard error	$<10^{-4}$	$<10^{-4}$	$<10^{-4}$	$<10^{-4}$	$<10^{-4}$	$<10^{-4}$	$<10^{-4}$	$<10^{-4}$
**Form Factor**								
Mean ± standard deviation	0.82± 0.01	0.82± 0.02	0.82± 0.03	0.82± 0.01	0.75± 0.03	0,73± 0.04	0.74± 0.04	0.75± 0.02
standard error	$<10^{-4}$	$<10^{-4}$	0.01	$<10^{-4}$	0.01	0.01	0.01	0.01

Literature

[1] Monneret, G.; Venet, F. Sepsis-Induced Immune Alterations Monitoring by Flow Cytometry as a Promising Tool for Individualized Therapy: MECHANISMS SUSTAINING SEPSIS-INDUCED IMMUNOSUPPRESSION. *Cytometry* **2016**, *90*, 376–386, doi:10.1002/cyto.b.21270.

[2] Yoon, J.; Jo, Y.; Kim, M.; Kim, K.; Lee, S.; Kang, S.-J.; Park, Y. Identification of Non-Activated Lymphocytes Using Three-Dimensional Refractive Index Tomography and Machine Learning. *Sci Rep* **2017**, *7*, 6654, doi:10.1038/s41598-017-06311-y.

[3] Hohlstein, P.; Gussen, H.; Bartneck, M.; Warzecha, K.T.; Roderburg, C.; Buendgens, L.; Trautwein, C.; Koch, A.; Tacke, F. Prognostic Relevance of Altered Lymphocyte Subpopulations in Critical Illness and Sepsis. *JCM* **2019**, *8*, 353, doi:10.3390/jcm8030353.

[4] Gawdat, K.; Legere, S.; Wong, C.; Myers, T.; Marshall, J.S.; Hassan, A.; Brunt, K.R.; Kienesberger, P.C.; Pulinilkunnil, T.; Legare, J.-F. Changes in Circulating Monocyte Subsets (CD16 Expression) and Neutrophil-to-Lymphocyte Ratio Observed in Patients Undergoing Cardiac Surgery. *Front. Cardiovasc. Med.* **2017**, *4*, doi:10.3389/fcvm.2017.00012.

[5] Hewing, B.; Ellerbroek, R.; Au, S.; Stangl, V.; Dreger, H.; Laule, M.; Grubitzsch, H.; Knebel, F.; Baumann, G.; Ludwig, A.; et al. Levels of Circulating Intermediate Monocytes Decrease after Aortic Valve Replacement in Patients with Severe Aortic Stenosis. *Thromb Haemost* **2017**, *117*, 2346–2355, doi:10.1160/TH17-05-0341.

[6] Suzuki, A.; Fukuzawa, K.; Yamashita, T.; Yoshida, A.; Sasaki, N.; Emoto, T.; Takei, A.; Fujiwara, R.; Nakanishi, T.; Yamashita, S.; et al. Circulating Intermediate CD14++CD16+monocytes Are Increased in Patients with Atrial Fibrillation and Reflect the Functional Remodelling of the Left Atrium. *Europace* **2016**, euv422, doi:10.1093/europace/euv422.

[7] Holmannova, D.; Kolackova, M.; Kunes, P.; Krejsek, J.; Mandak, J.; Andrys, C. Impact of Cardiac Surgery on the Expression of CD40, CD80, CD86 and HLA-DR on B Cells and Monocytes. *Perfusion* **2016**, *31*, 391–400, doi:10.1177/0267659115612905.

[8] Greco, M.; Mazzei, A.; Palumbo, C.; Verri, T. Flow Cytometric Analysis of Monocytes Polarization and Reprogramming from Inflammatory to Immunosuppressive Phase during Sepsis. 14.

[9] Coillie S; Wiernicki B; Xu J Molecular and Cellular Functions of CTLA-4. *Adv Exp Med Biol.* **2020**, 1248:7-32, doi:10.1007/978-981-15-3266-5_2.

[10] Quadrini, K.J.; Patti-Diaz, L.; Maghsoudlou, J.; Cuomo, J.; Hedrick, M.N.; McCloskey, T.W. A Flow Cytometric Assay for HLA-DR Expression on Monocytes Validated as a Biomarker for Enrollment in Sepsis Clinical Trials. *Cytometry* **2021**, *100*, 103–114, doi:10.1002/cyto.b.21987.

[11] Albertsmeier, M.; Prix, N.J.; Winter, H.; Bazhin, A.; Werner, J.; Angele, M.K. Monocyte-Dependent Suppression of T-Cell Function in Postoperative Patients and Abdominal Sepsis. *Shock* **2017**, *48*, 651–656, doi:10.1097/SHK.0000000000000924.

Figure S1. Representative bright-field images of isolated lymphocytes (A) and monocytes (B). Attached or destroyed cells were excluded from measurement (marked with black arrows in S1A and S1B). Monocytes that were tethered by platelets were also excluded due to a loss of spheric cell property (platelet-monocyte complex marked with black triangle)

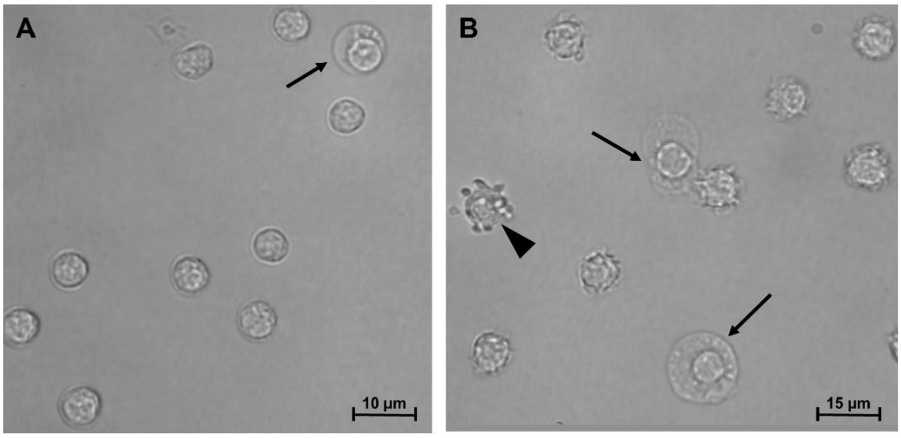

Figure S2. Density scatterplots of biophysical cell parameters during the perioperative course with a uniform presentation of the cell volume against the other parameters corresponding to Fig. 4 A, B and C.

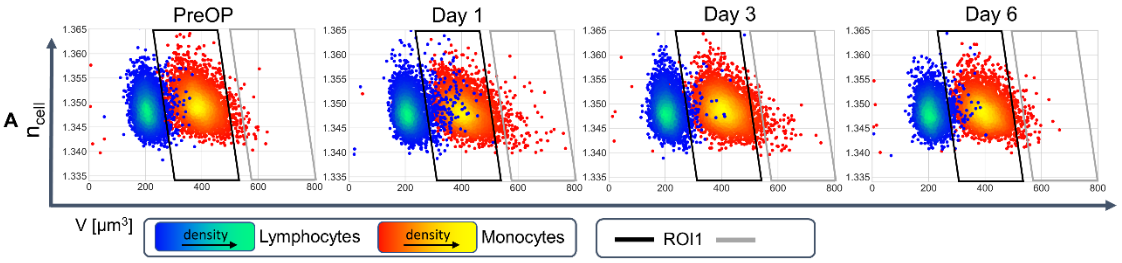

Figure S3. Standard deviations of the average values in Fig. 5. Each data point represents the standard deviation of the average value from a fraction of 150 cells that were analyzed per time point and individual patient corresponding to Fig. 5. In analogy to an increased density of scattering on day 1 post-surgery shown in Fig. 4, the standard deviation of both lymphocyte and monocyte volume is significantly higher on first postoperative day (for monocytes and lymphocytes between preOP and d1 $p < 0.0001$). For both cell types the standard deviation decreases over the next measured days. While lymphocytes immediately decrease on the third postoperative day significantly ($p = 0.0067$), monocytes decrease significantly later (on the sixth day, $p = 0.0001$). There is a significant increase of lymphocyte dry mass on the first postoperative ($p = 0.0006$) and again a significant decrease on day 3 ($p = 0.0004$) while there is no significance regarding monocyte dry mass.
The standard deviation of the monocyte refractive index decreases significantly between PreOP and day 3 ($p = 0.0092$) and between PreOP and day 6 ($p = 0.0174$) while there is no significant difference between PreOP and day 1 post-surgery. * $p < 0.05$; ** $p < 0.01$, *** $p < 0.001$.

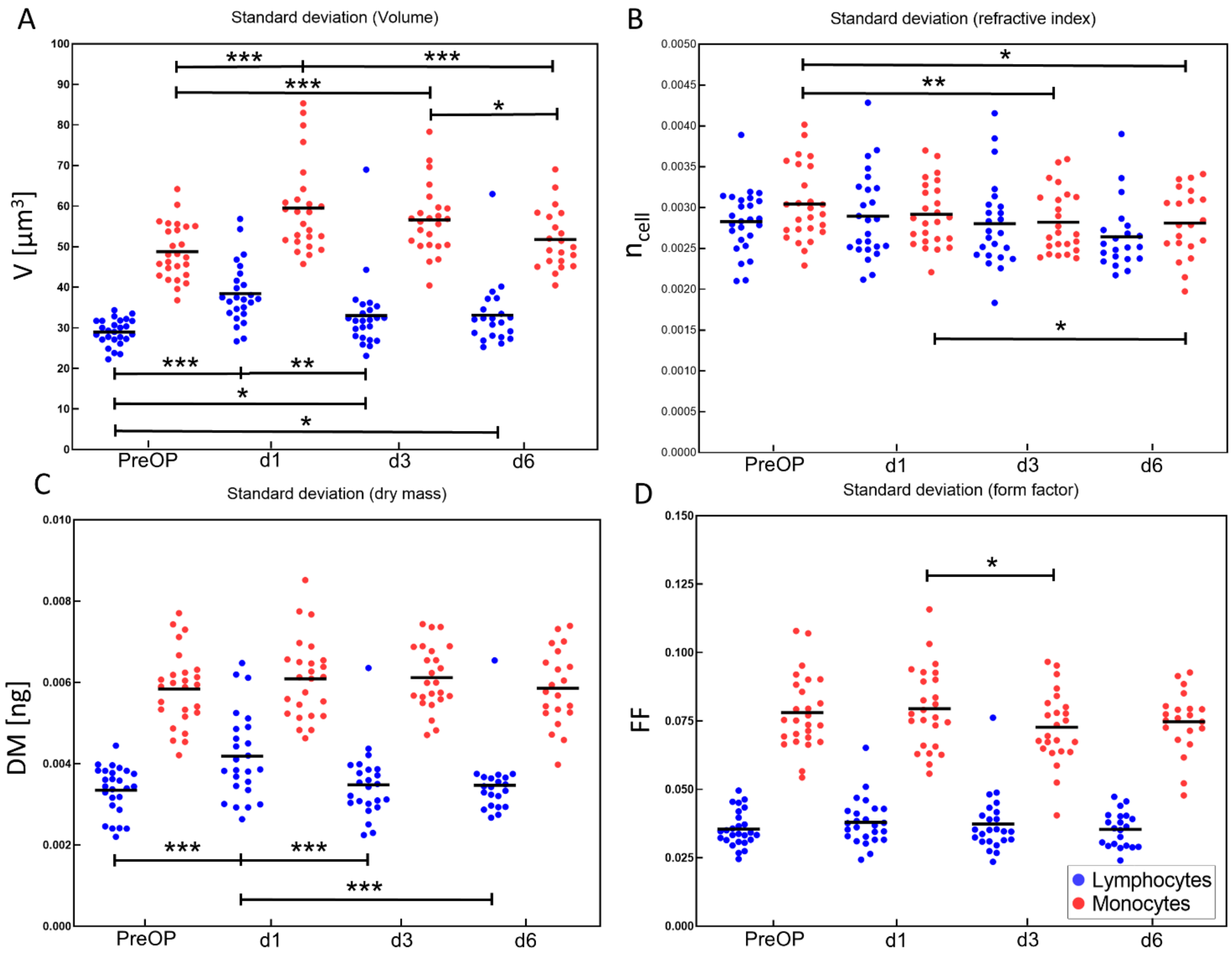

Figure S4. Fig. S4 shows lymphocyte volume changes for individual patients during the perioperative course for the three subgroups identified in Fig. 6D. No significant changes in monocytes were detected.
Each data point represents the average value from a fraction of 150 cells that were analyzed per time point and individual patient. Data points were connected to track the course of individual patients. Significant changes were detected in lymphocyte subgroups with complicated and regular perioperative course (S4A), without epinephrine treatment (S4B, lower row) as well as in patients with high and low CRP level (S4C). Individual patients in the group with high CRP (> 14) showed an increase of volume one day post-surgery (p = 0.03). Non-uniform changes in lymphocyte volume were observed for individual patients within the group treated with epinephrine while the mean values were constant (not significant). Lymphocyte volume of patients with regular courses, low CRP level and without epinephrine treatment showed perioperatively similar trends with a high increase on day 1 and a significant decrease on day 3. The individual values of the main fraction of patients were observed to follow the tendency of the mean values. Note that discontinued lines on day 3 indicate patients that left the hospital before a 4th measurement while one patient died after one day post-surgery (discontinued lines on day 1 in S4B and S4C). These patient courses represent outliers from the tendencies of the mean values. * p < 0.05; ** p < 0.01, *** p < 0.001.
Individual courses of lymphocyte volume for three patient subgroups corresponding to Fig. 6D

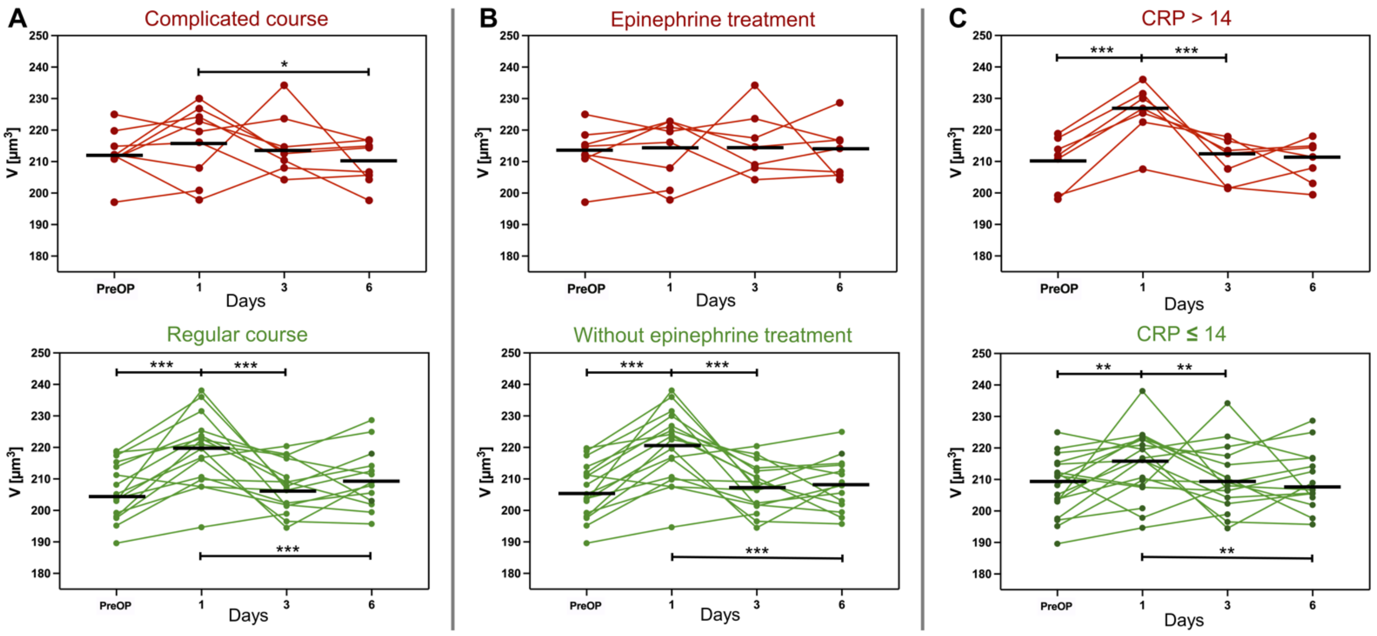

Figure S5. Further comparisons of parameters between the three subgroups corresponding to Fig. 6D. Comparison of parameters at single measurement days (PreOP, d1, d3, d6) between regular and complicated postoperative course (A-C) as well as with or without epinephrine treatment (D) as well. The number of T cells (CD3*) and T helper cells (CD4*) was significantly lower in the group with complicated courses at day 1, day 3 and day 6 (A, B). In contrast, the number of apoptotic cells was increased PreOP in patients with complicated course (C). Platelets decreased significantly on day 1 and highly significantly on day 6 due to epinephrine treatment (D).

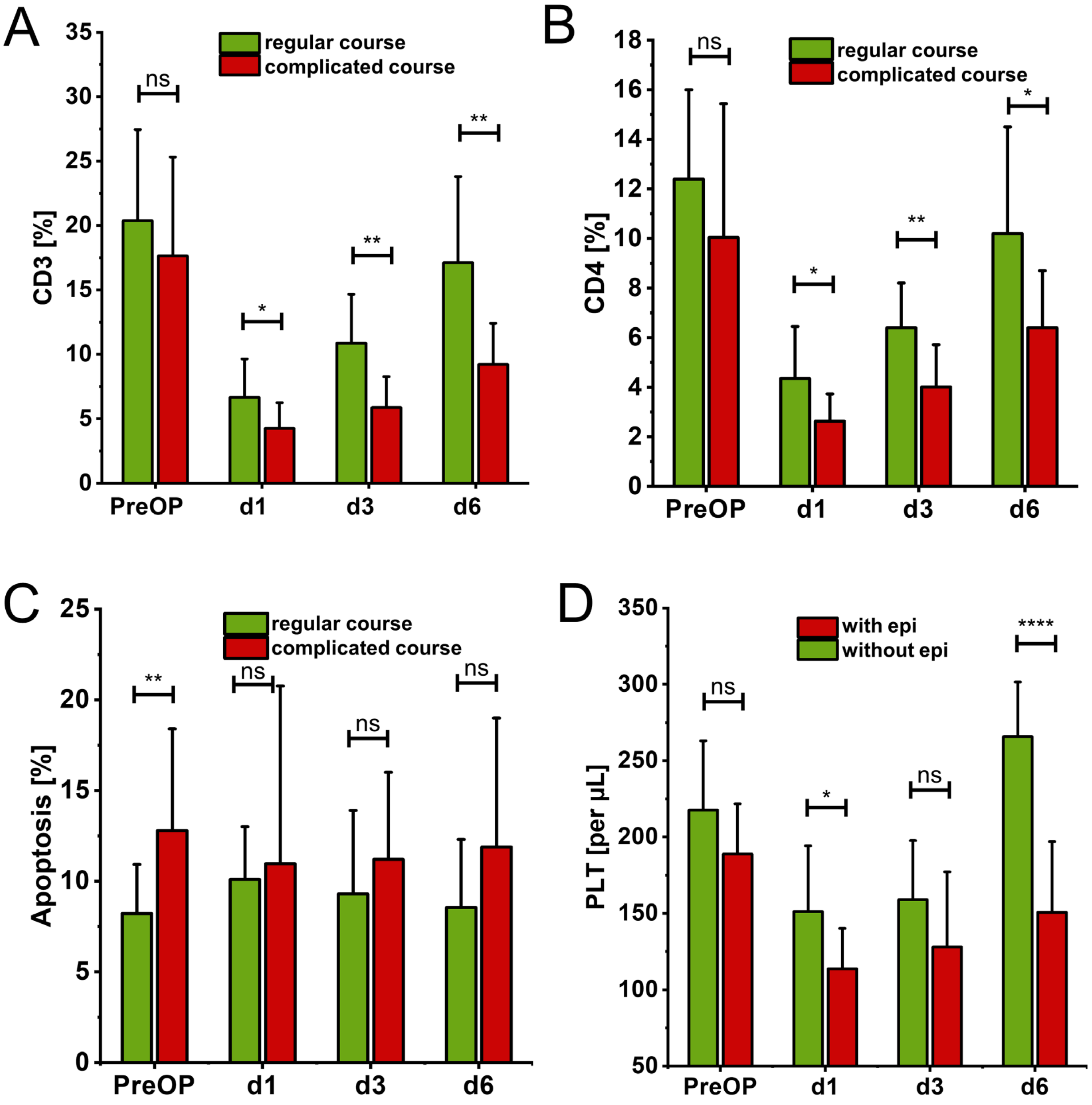

Figure S6. Representative correlation plot for ∆V-L vs. ∆CD19abs corresponding to Table 1. Data from individual patients are presented by numbers (from patient 1 to patient 25)

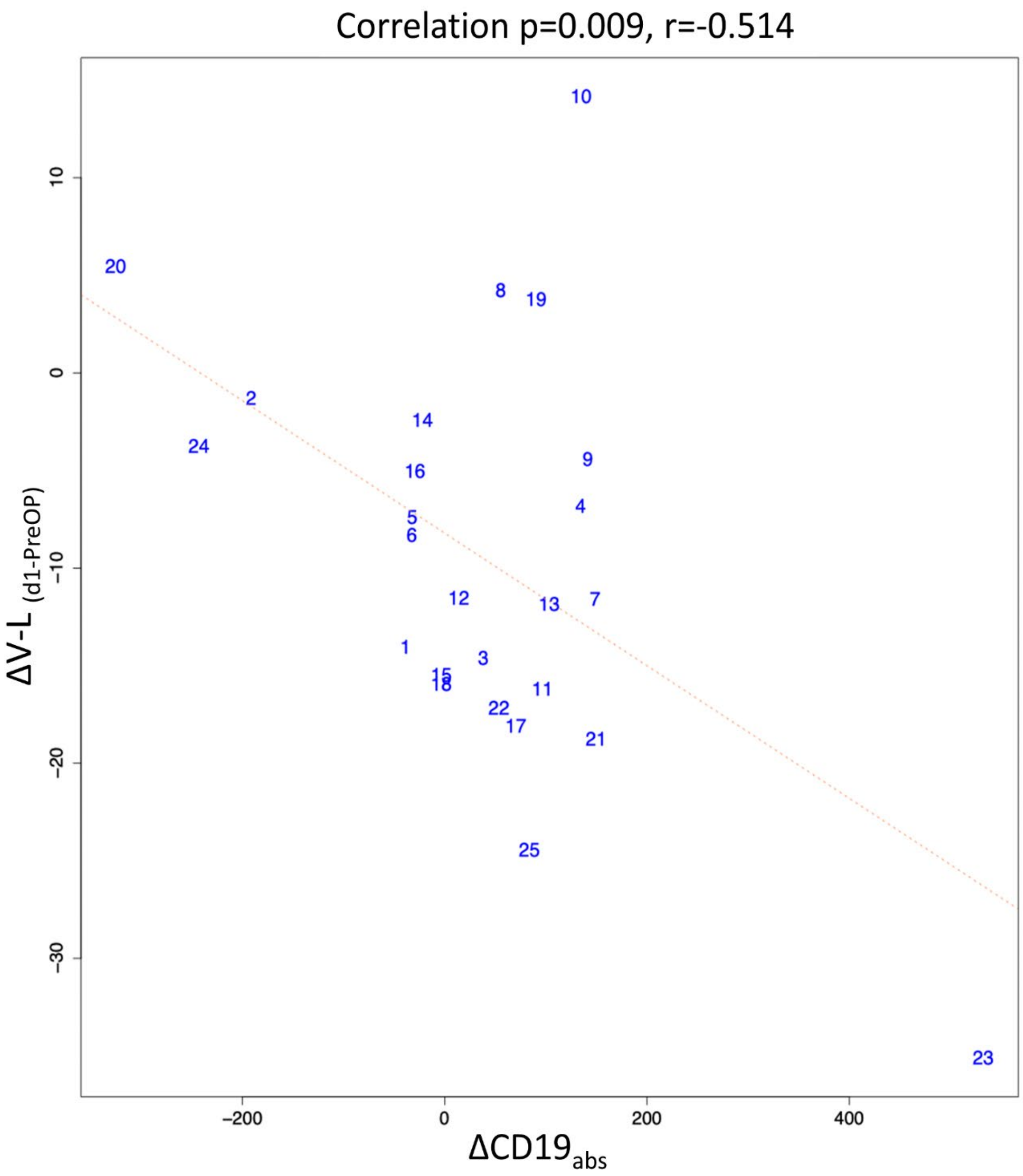

Supplement: Supplementary file 1 [file cells-11-00755-s001.zip › Supplement_S1.pdf]
