# Supplementary material for: Digital Holographic Microscopy for Label-Free Detection of Leukocyte Alternations Associated with Perioperative Inflammation after Cardiac Surgery
Source: Cells. 2022 Feb 21;11(4):755. doi: 10.3390/cells11040755 (PMC8869820; doi:10.3390/cells11040755)
Supplement: Supplementary file 1 [file cells-11-00755-s001.zip › Supplement_S3.pdf]

Supplement S3

Corresponding to Fig. 4

Density scatterplots of all individual patients (n=25)

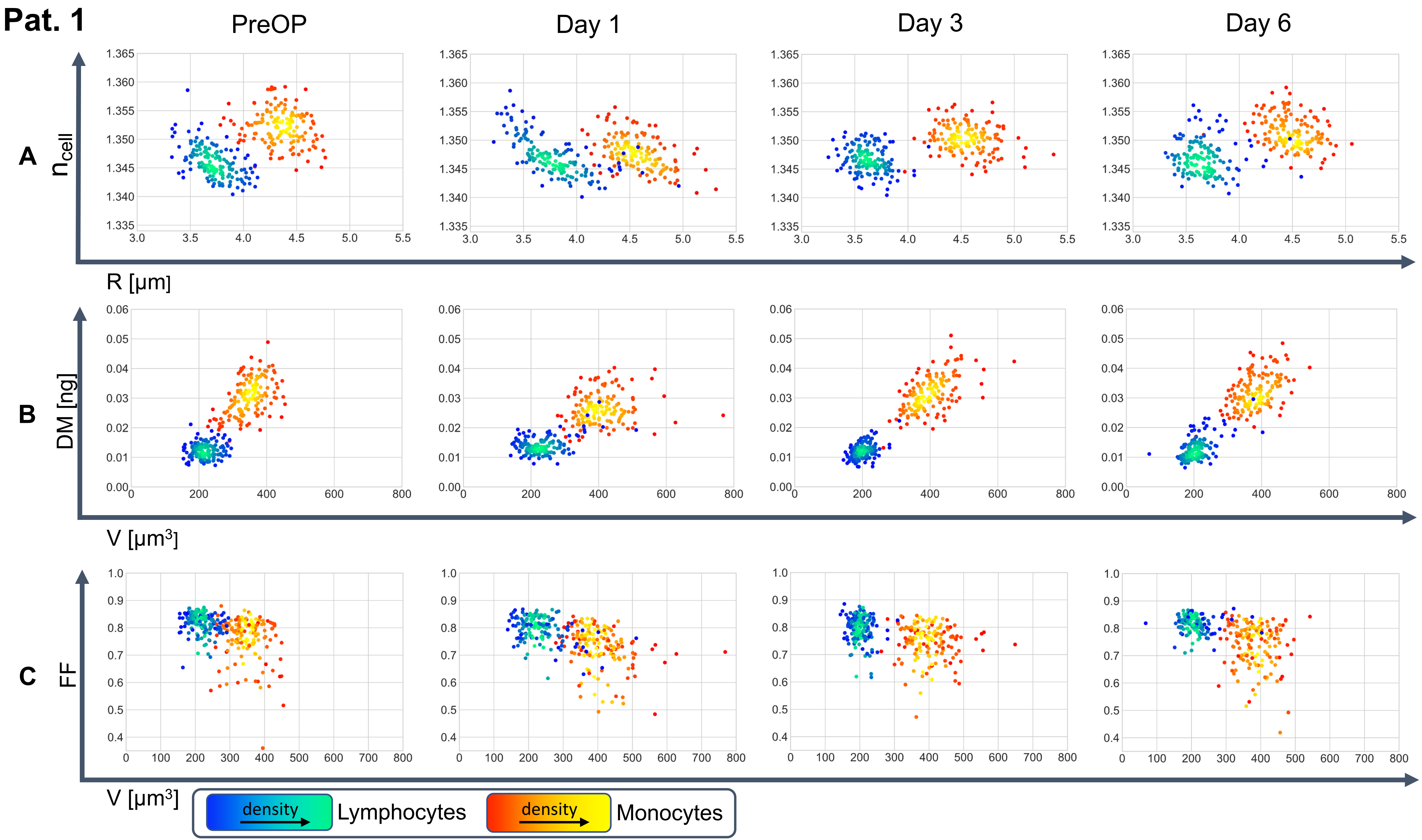

Pat. 1
PreOP
Day 1
Day 3
Day 6
A
n_{cell}
R [µm]
B
DM [ng]
V [µm³]
C
FF
V [µm³]
density
Lymphocytes
density
Monocytes

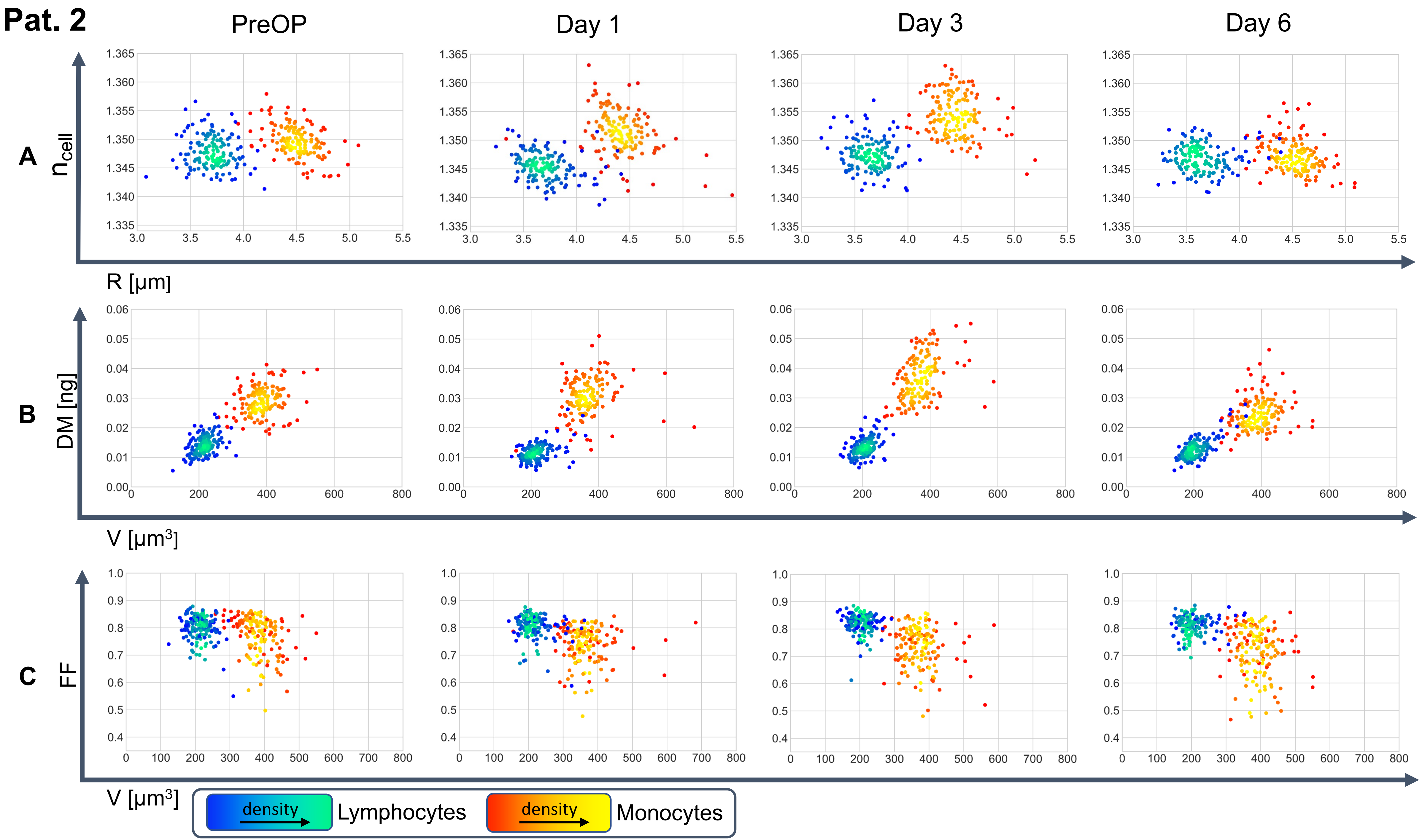
Pat. 2
PreOP
Day 1
Day 3
Day 6
A
n_{cell}
R [µm]
B
DM [ng]
V [µm³]
C
FF
V [µm³]
density
Lymphocytes
density
Monocytes

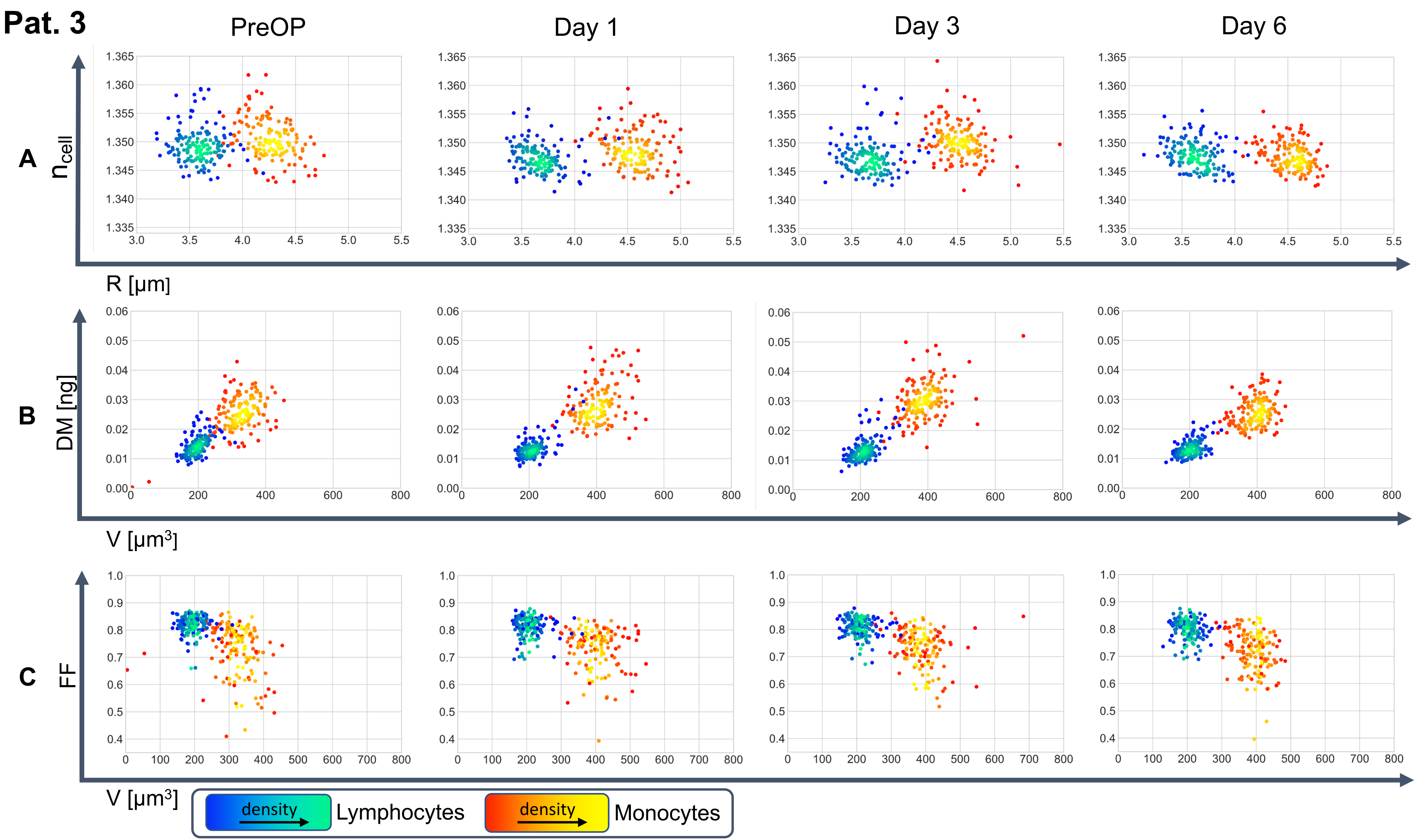

Pat. 3
PreOP
Day 1
Day 3
Day 6
A
n_{cell}
R [µm]
B
DM [ng]
V [µm³]
C
FF
V [µm³]
density
Lymphocytes
density
Monocytes

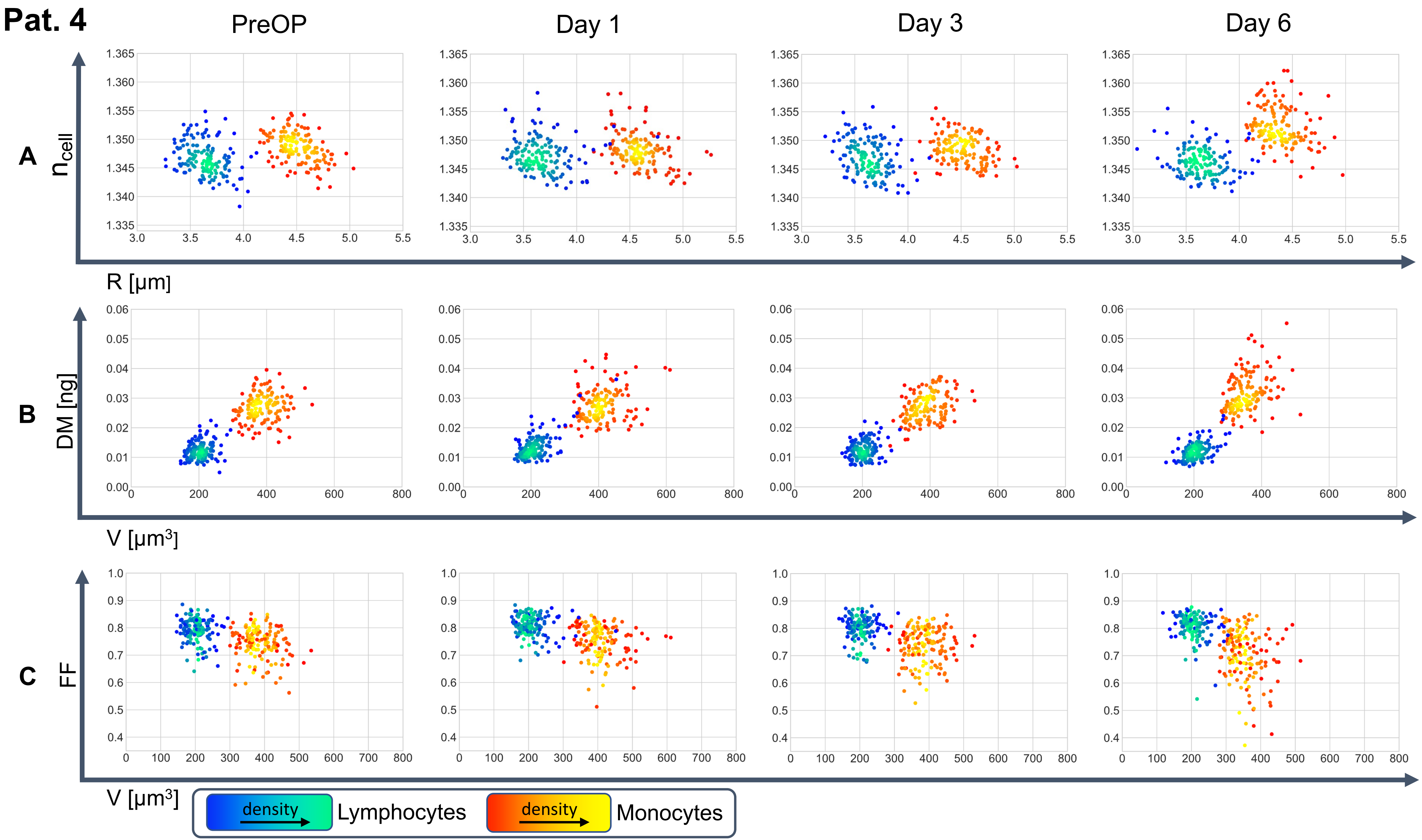
Pat. 4
PreOP
Day 1
Day 3
Day 6
A
n_{cell}
R [µm]
B
DM [ng]
V [µm³]
C
FF
V [µm³]
density
Lymphocytes
density
Monocytes

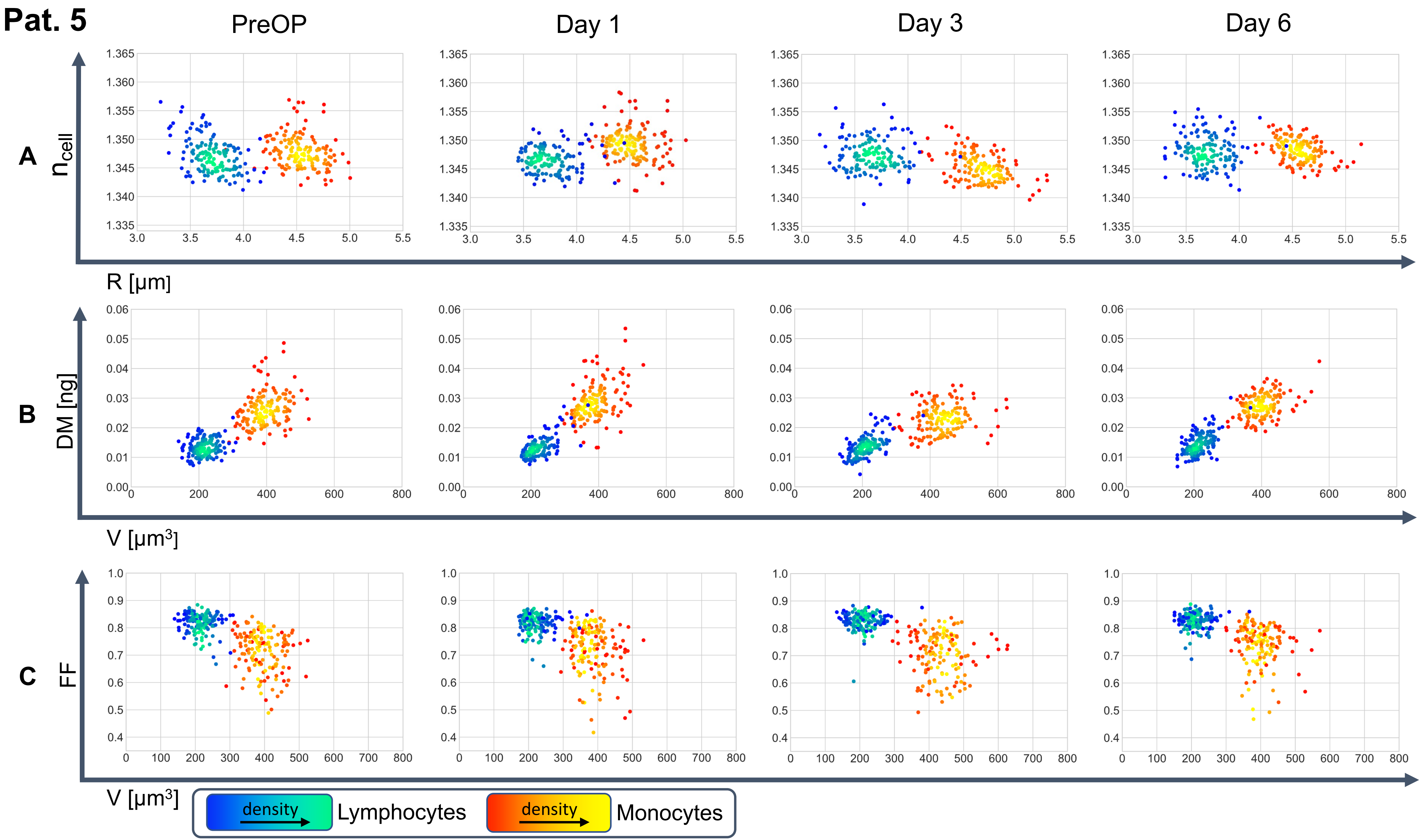

Pat. 5
PreOP
Day 1
Day 3
Day 6
A
n cell
R [µm]
B
DM [ng]
V [µm³]
C
FF
V [µm³]
density
Lymphocytes
density
Monocytes

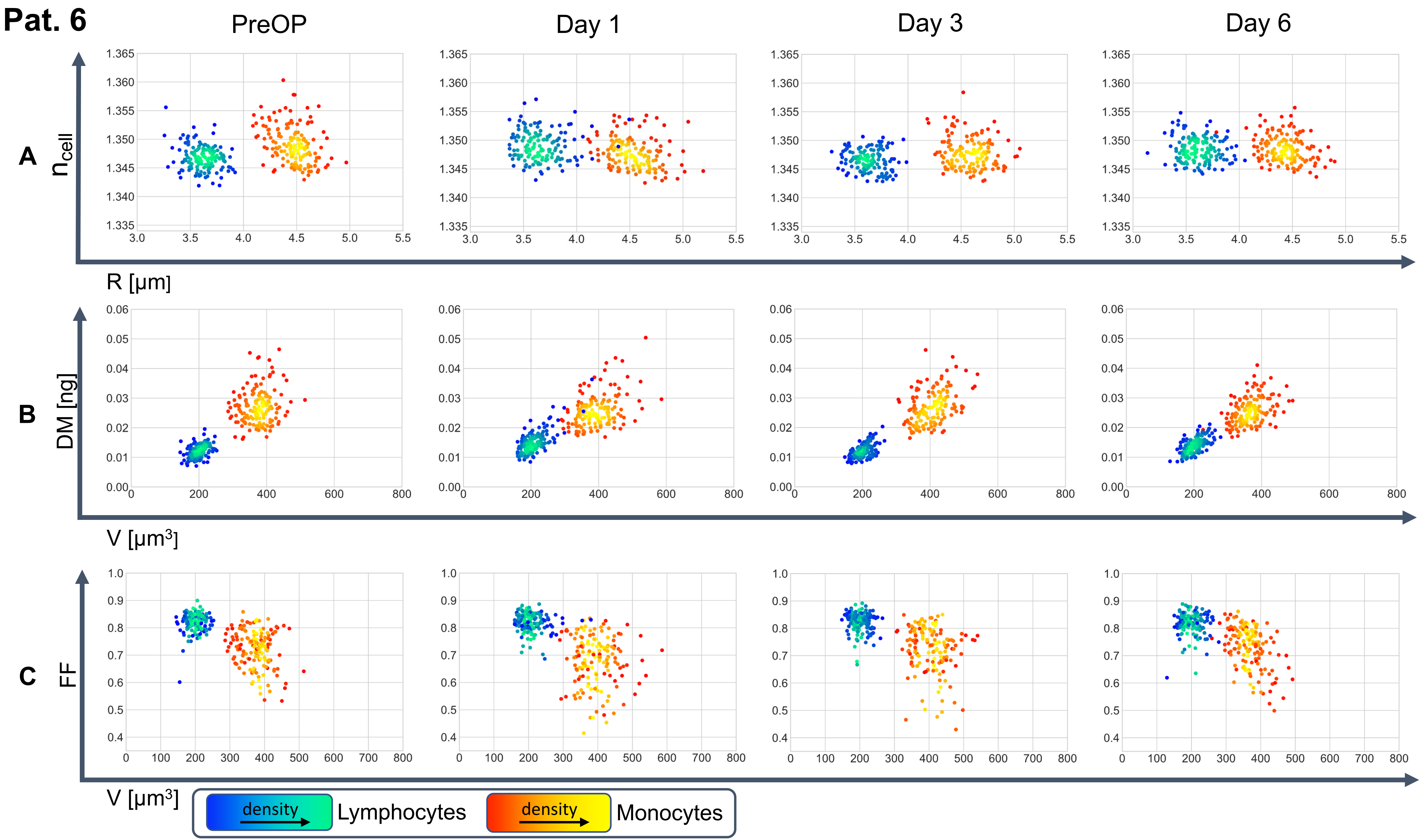
Pat. 6
PreOP
Day 1
Day 3
Day 6
A
n_{cell}
R [µm]
B
DM [ng]
V [µm³]
C
FF
V [µm³]
density
Lymphocytes
density
Monocytes

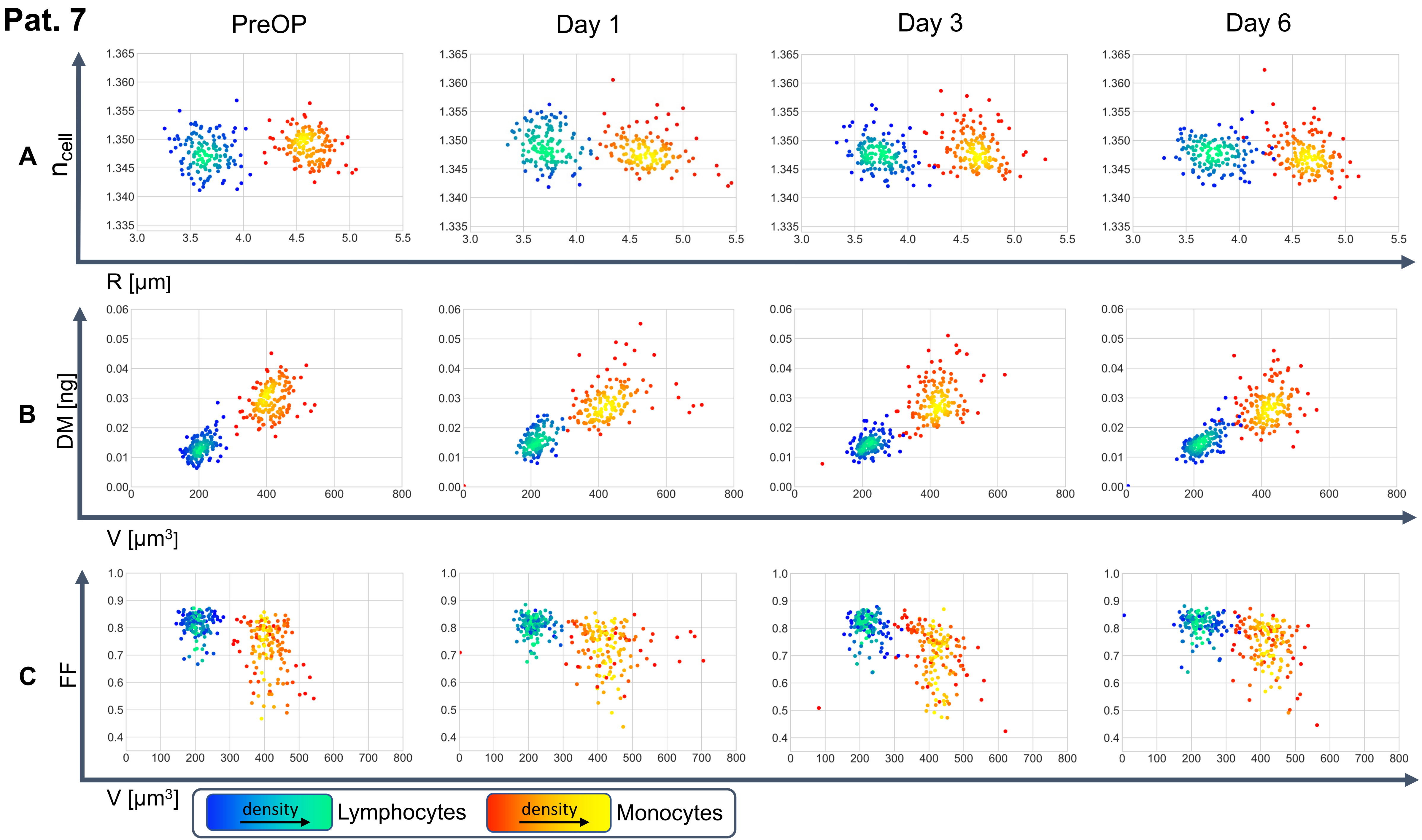
Pat. 7
PreOP
Day 1
Day 3
Day 6
A
B
C
n_cell
R [µm]
DM [ng]
V [µm³]
FF
V [µm³]
density
Lymphocytes
density
Monocytes

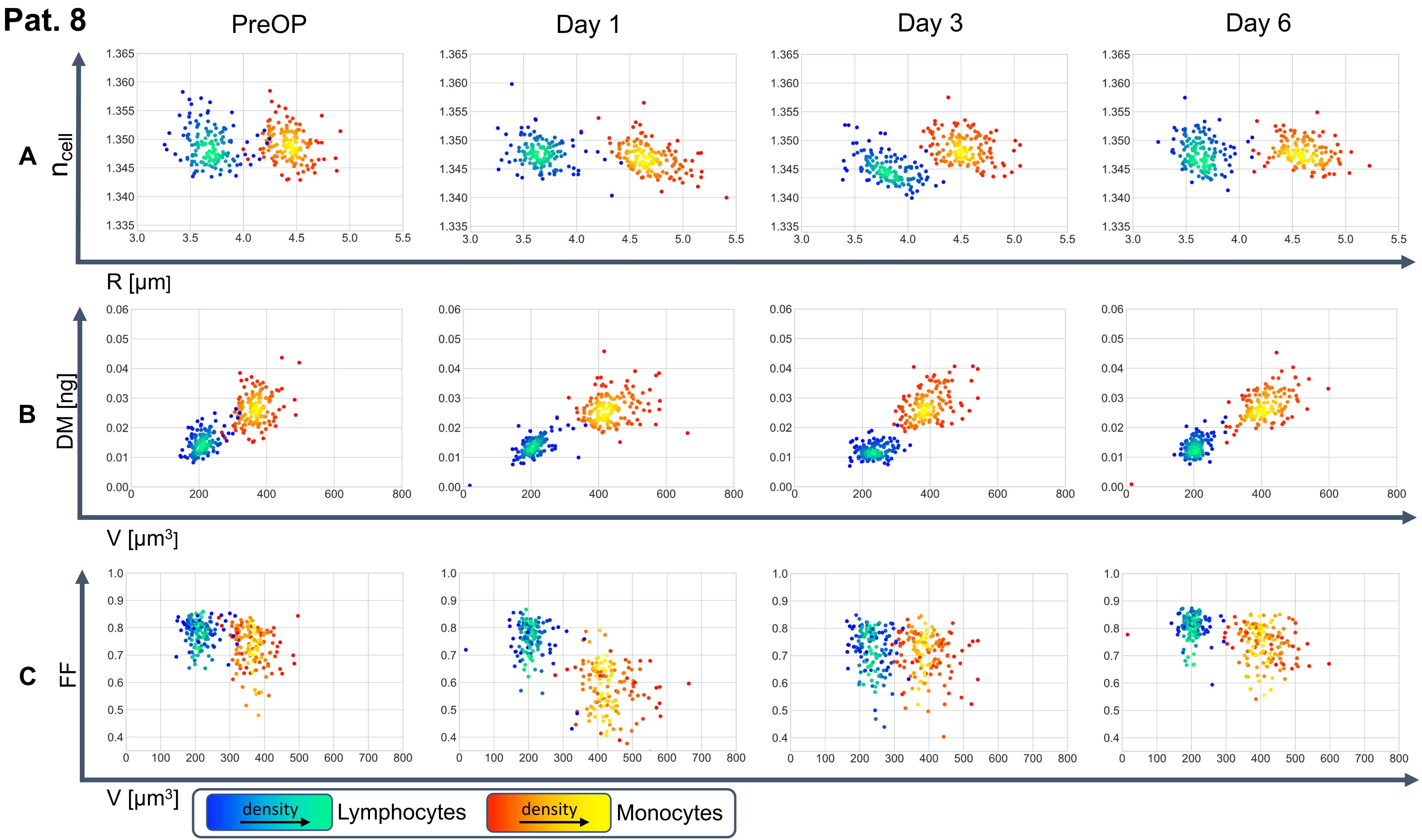

Pat. 8
PreOP
Day 1
Day 3
Day 6
A
n_cell
R [μm]
B
DM [ng]
V [μm³]
C
FF
V [μm³]
density
Lymphocytes
density
Monocytes

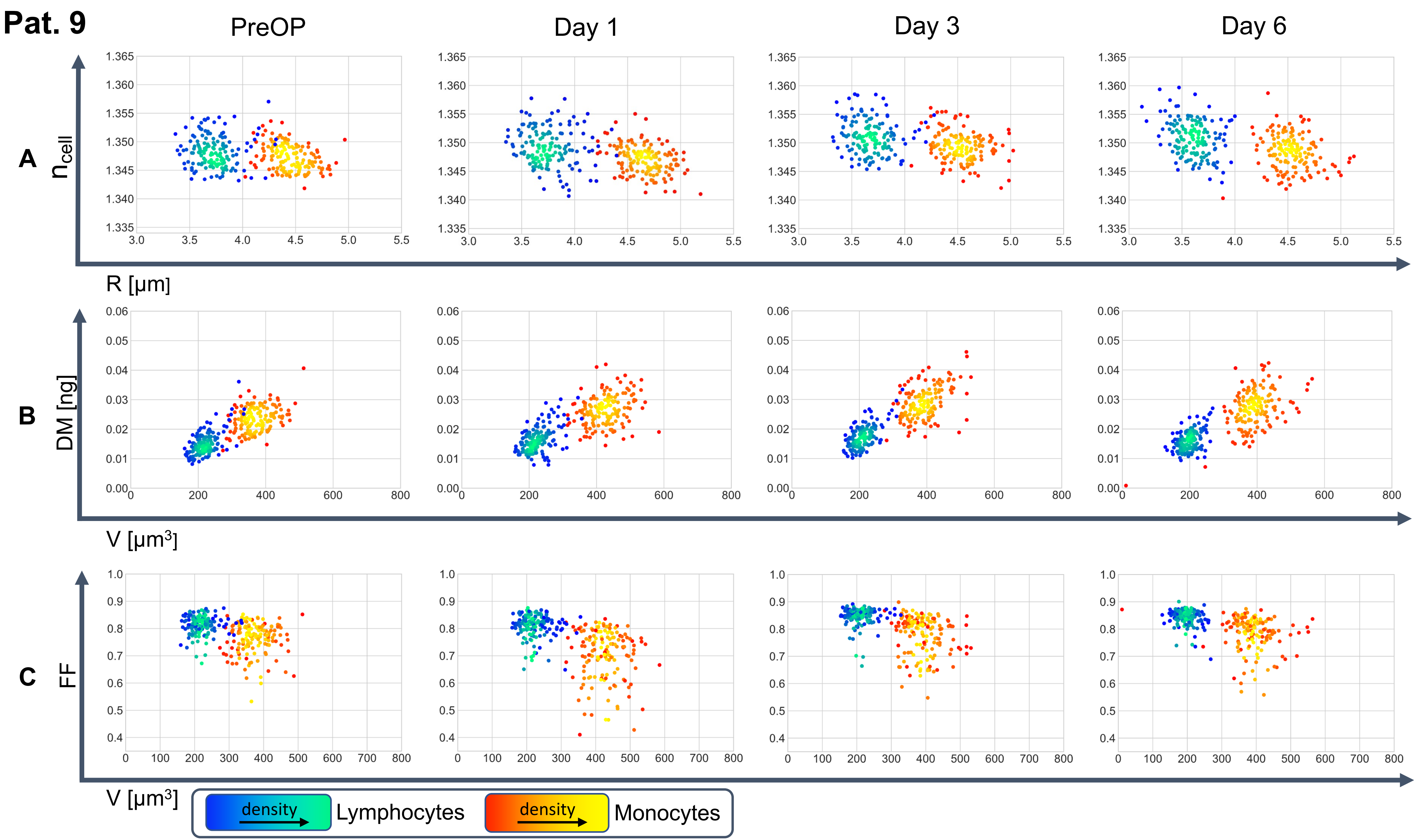

Pat. 9
PreOP
Day 1
Day 3
Day 6
A
n_{cell}
R [µm]
B
DM [ng]
V [µm³]
C
FF
V [µm³]
density
Lymphocytes
density
Monocytes

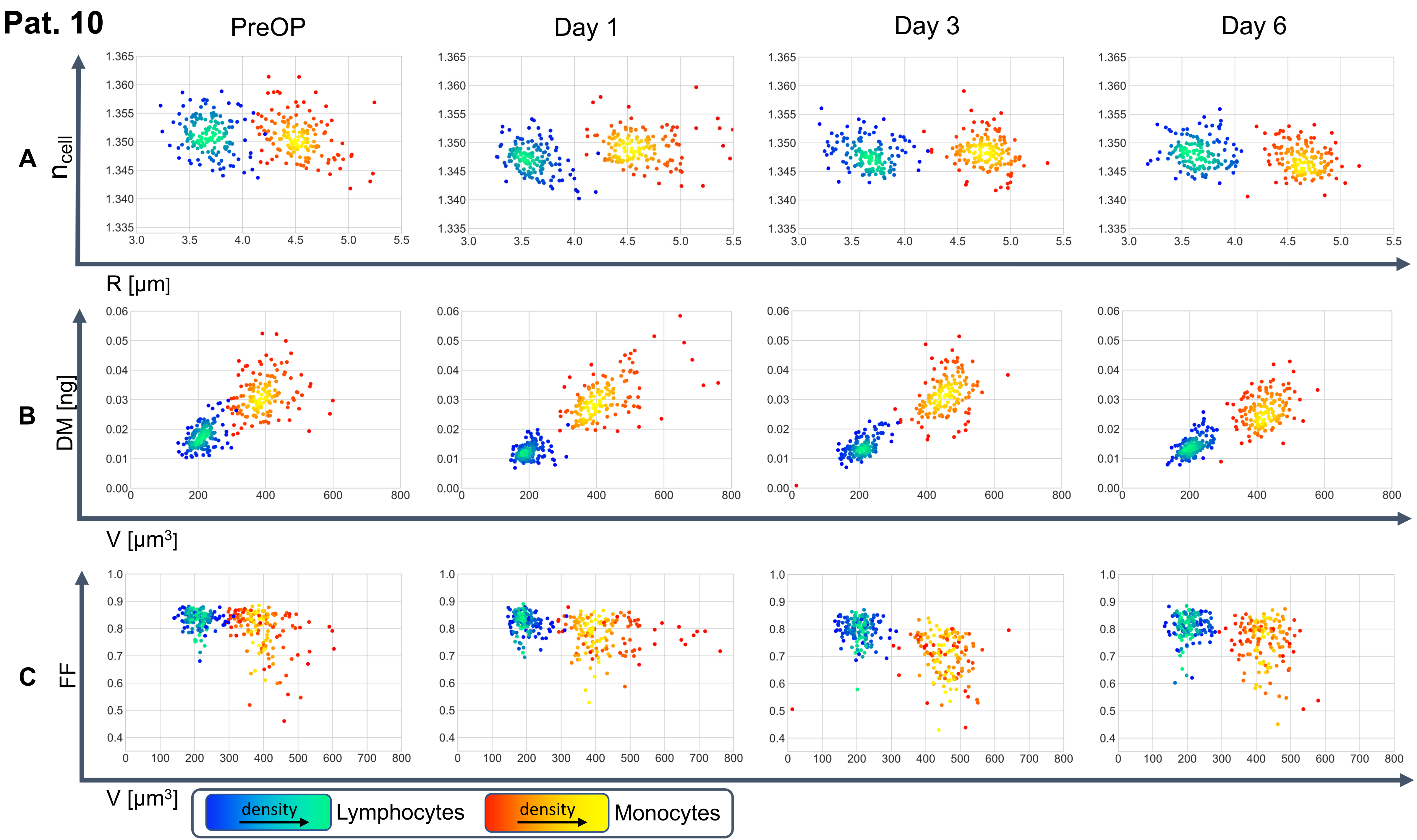

Pat. 10
PreOP
Day 1
Day 3
Day 6
A
n_{cell}
R [µm]
B
DM [ng]
V [µm³]
C
FF
V [µm³]
density
Lymphocytes
density
Monocytes

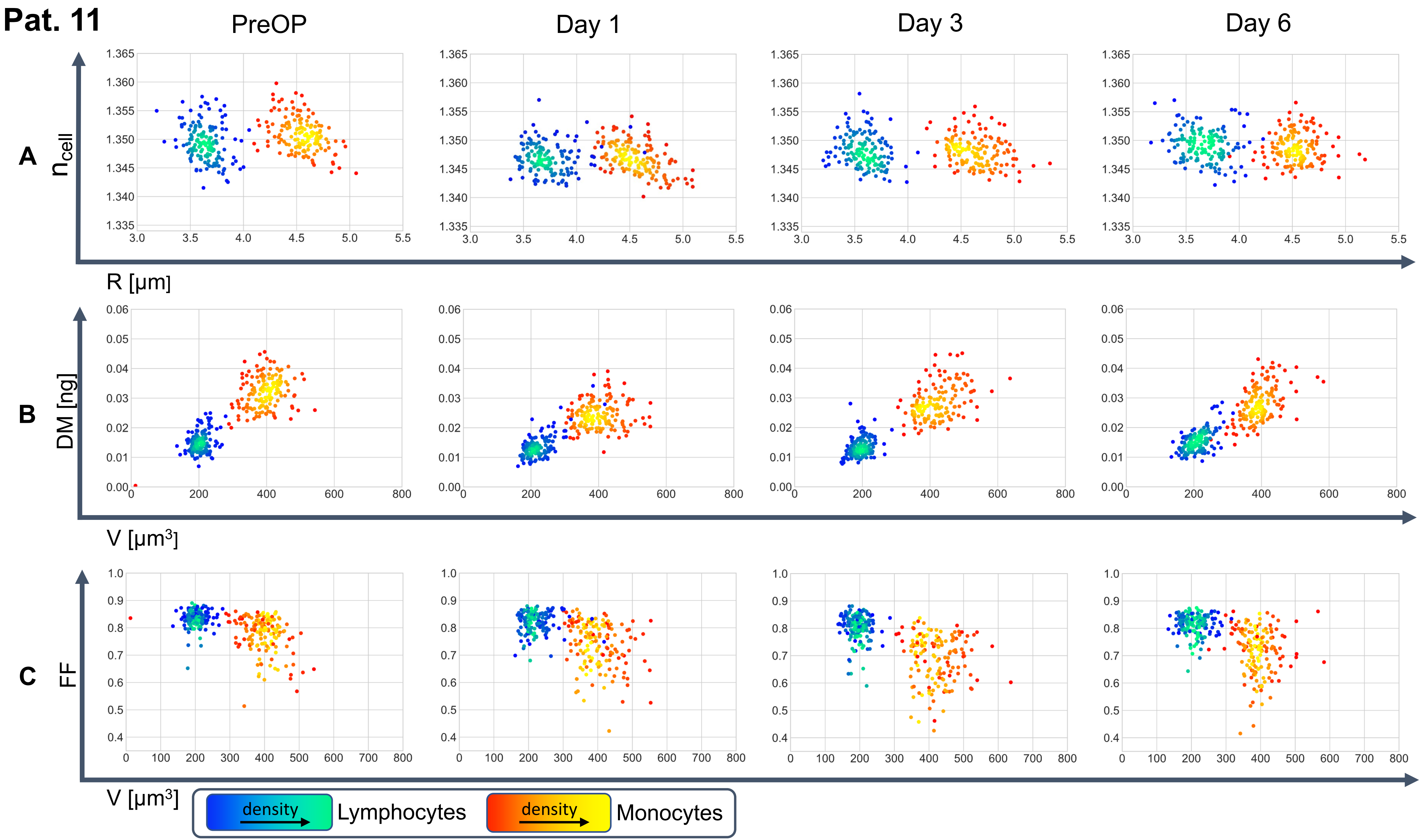
Pat. 11
PreOP
Day 1
Day 3
Day 6
A
n_cell
R [μm]
B
DM [ng]
V [μm³]
C
FF
V [μm³]
density
Lymphocytes
density
Monocytes

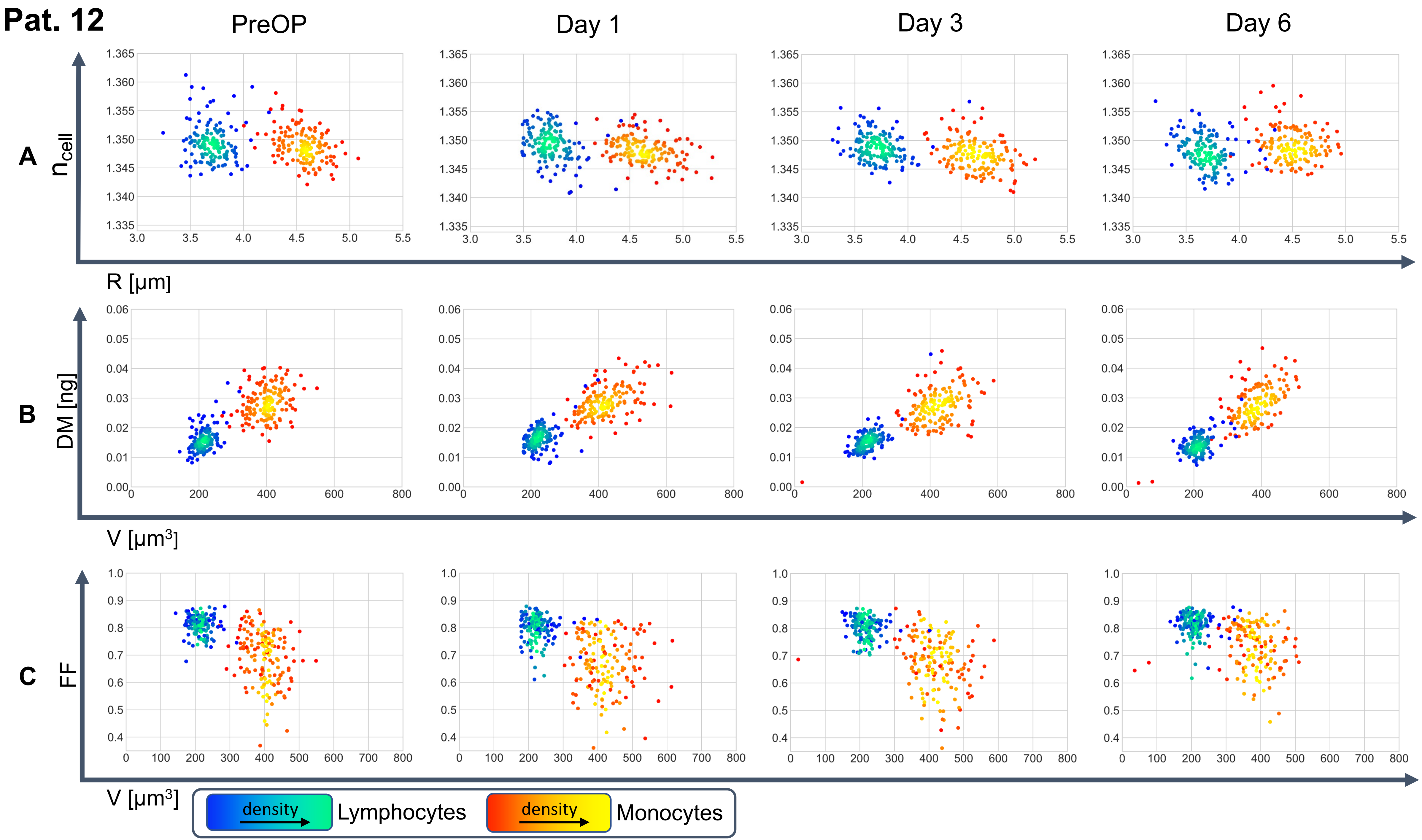
Pat. 12
PreOP
Day 1
Day 3
Day 6
A
n_cell
R [μm]
B
DM [ng]
V [μm³]
C
FF
V [μm³]
density
Lymphocytes
density
Monocytes

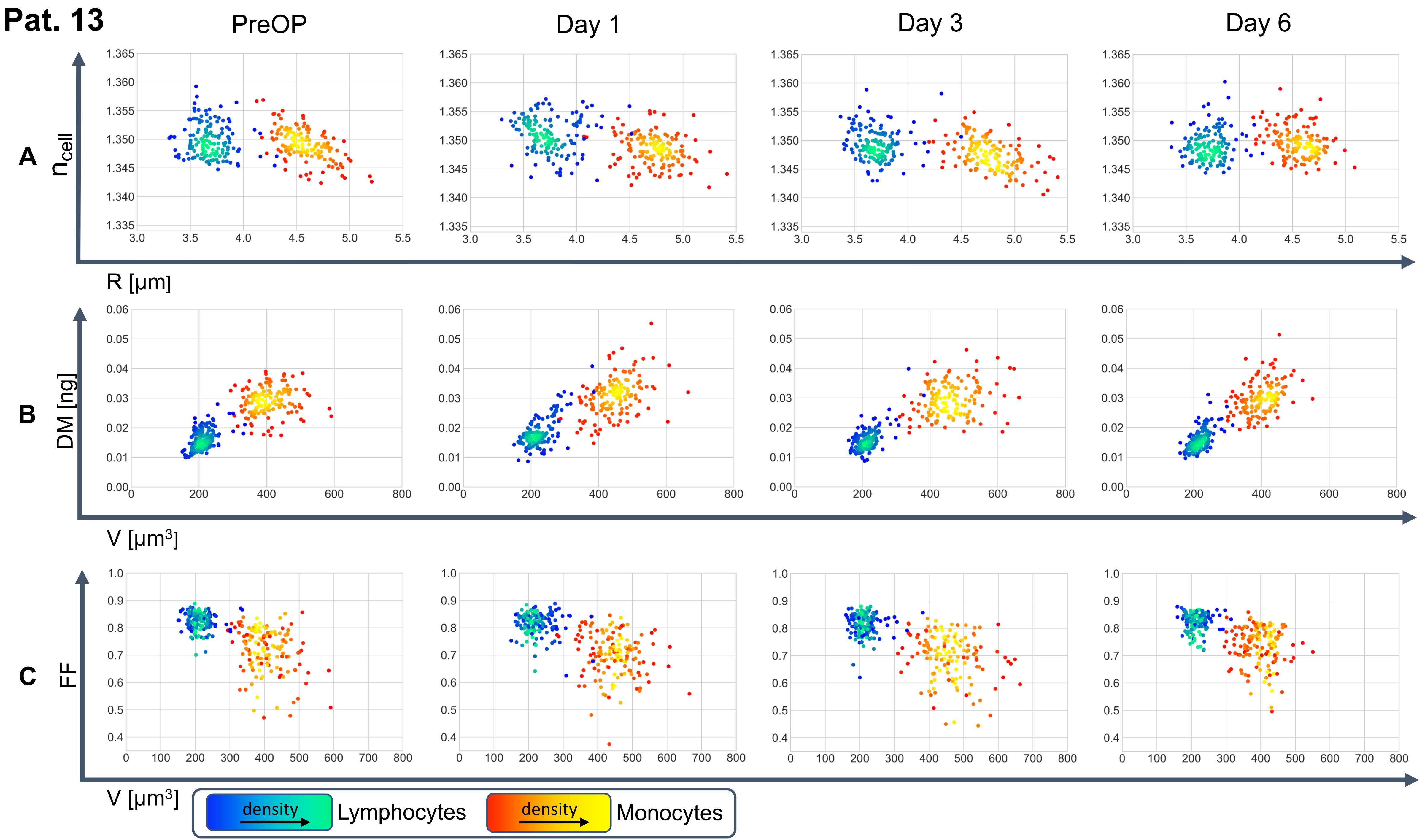

Pat. 13
PreOP
Day 1
Day 3
Day 6
A
n_cell
R [µm]
B
DM [ng]
V [µm³]
C
FF
V [µm³]
density
Lymphocytes
density
Monocytes

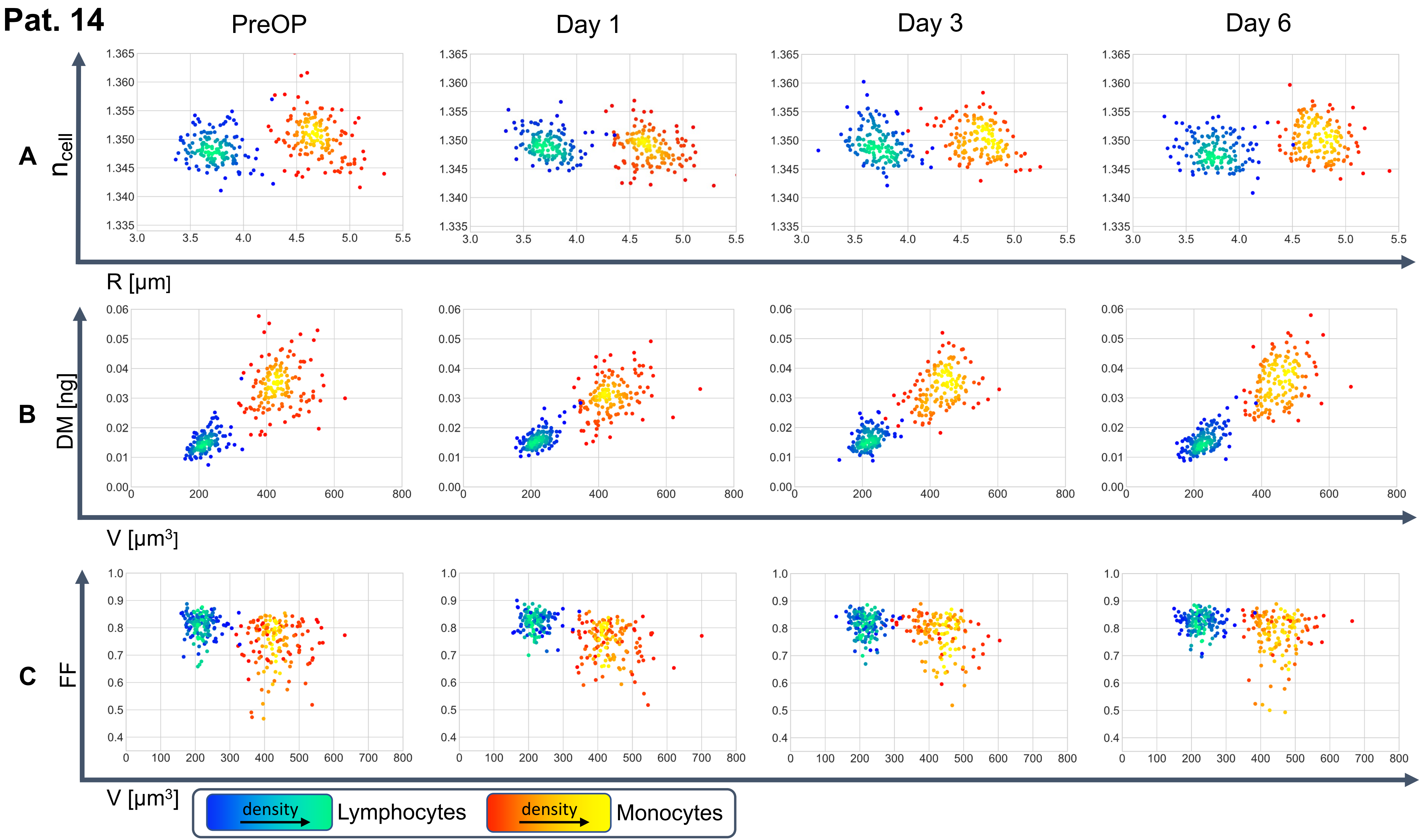

Pat. 14
PreOP
Day 1
Day 3
Day 6
A
n_{cell}
R [µm]
B
DM [ng]
V [µm³]
C
FF
V [µm³]
density
Lymphocytes
density
Monocytes

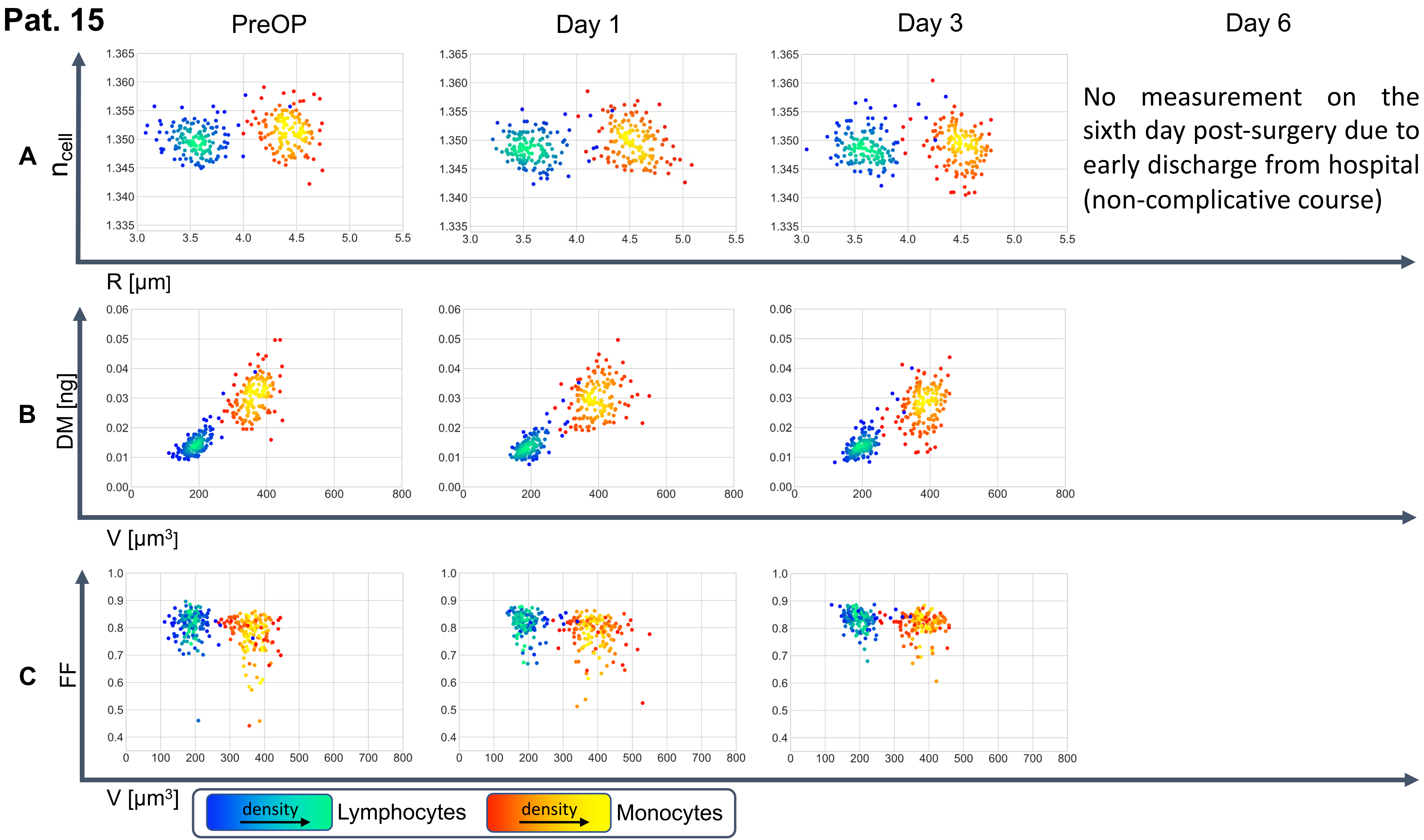
Pat. 15
PreOP
Day 1
Day 3
Day 6
No measurement on the sixth day post-surgery due to early discharge from hospital (non-complicative course)
A
ncell
R [µm]
B
DM [ng]
V [µm3]
C
FF
V [µm3]
density
Lymphocytes
density
Monocytes

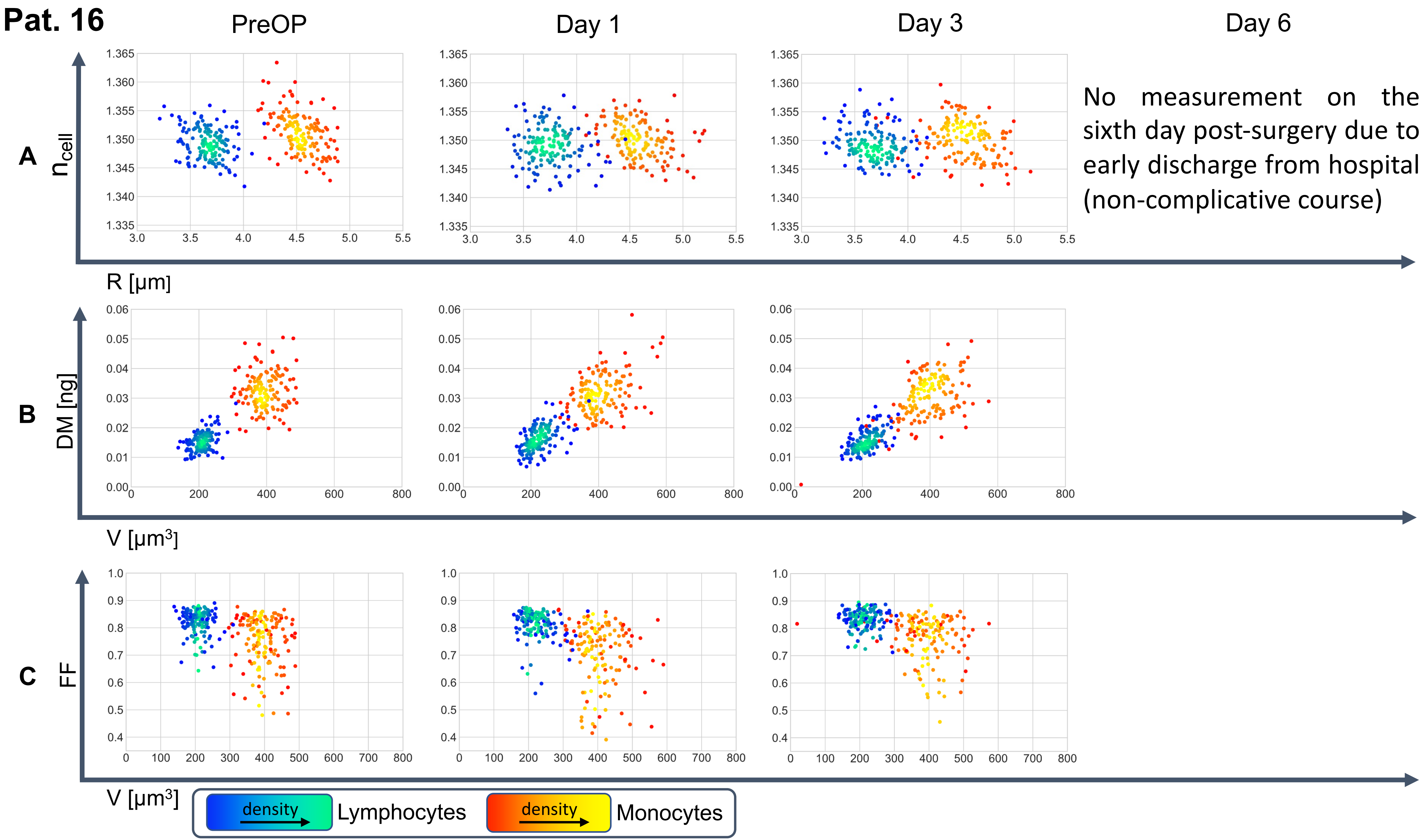

Pat. 16
PreOP
Day 1
Day 3
Day 6
No measurement on the sixth day post-surgery due to early discharge from hospital (non-complicative course)
A
n_cell
R [µm]
B
DM [ng]
V [µm³]
C
FF
V [µm³]
density
Lymphocytes
density
Monocytes

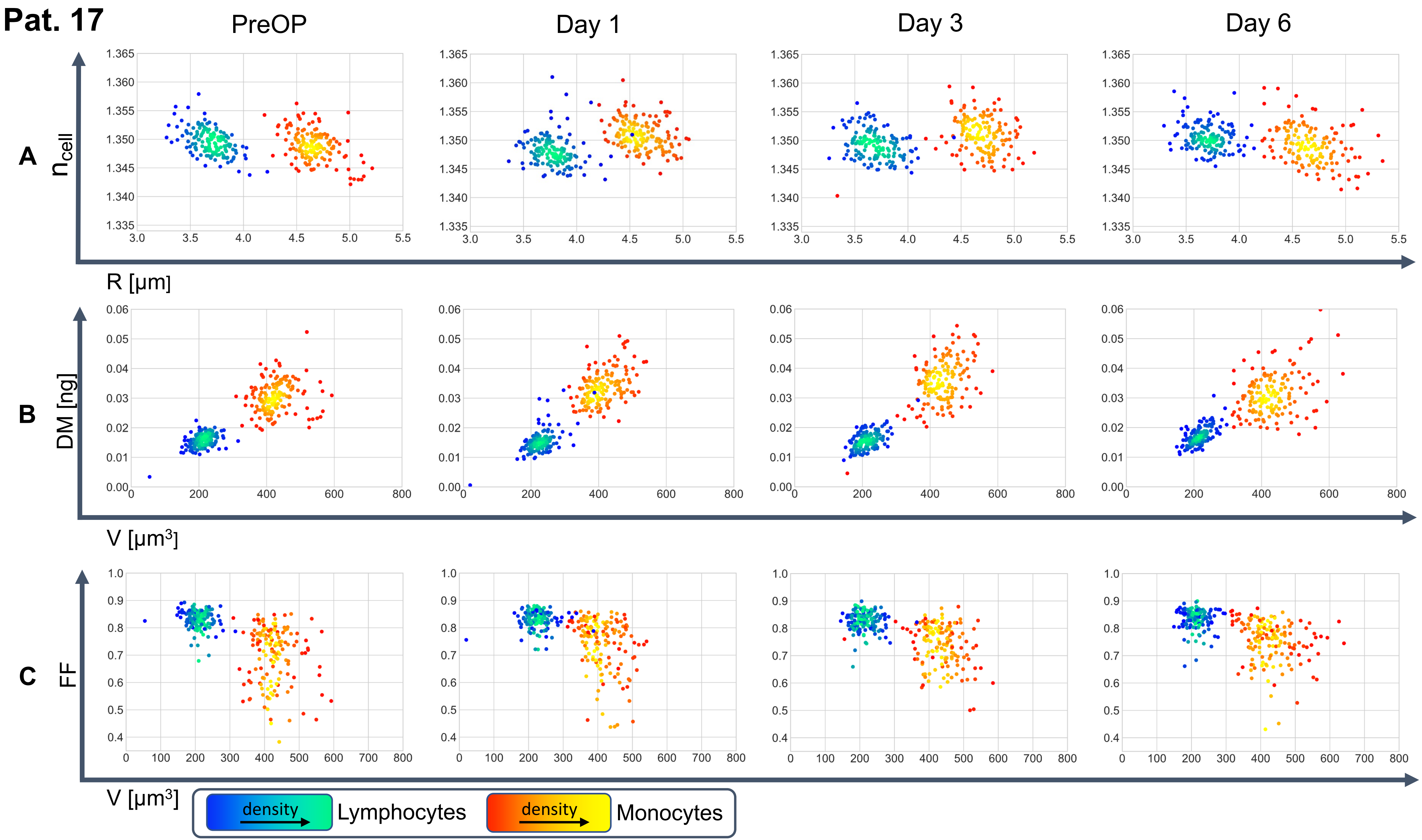

Pat. 17
PreOP
Day 1
Day 3
Day 6
A
B
C
n_cell
R [µm]
DM [ng]
V [µm³]
FF
V [µm³]
density
Lymphocytes
density
Monocytes

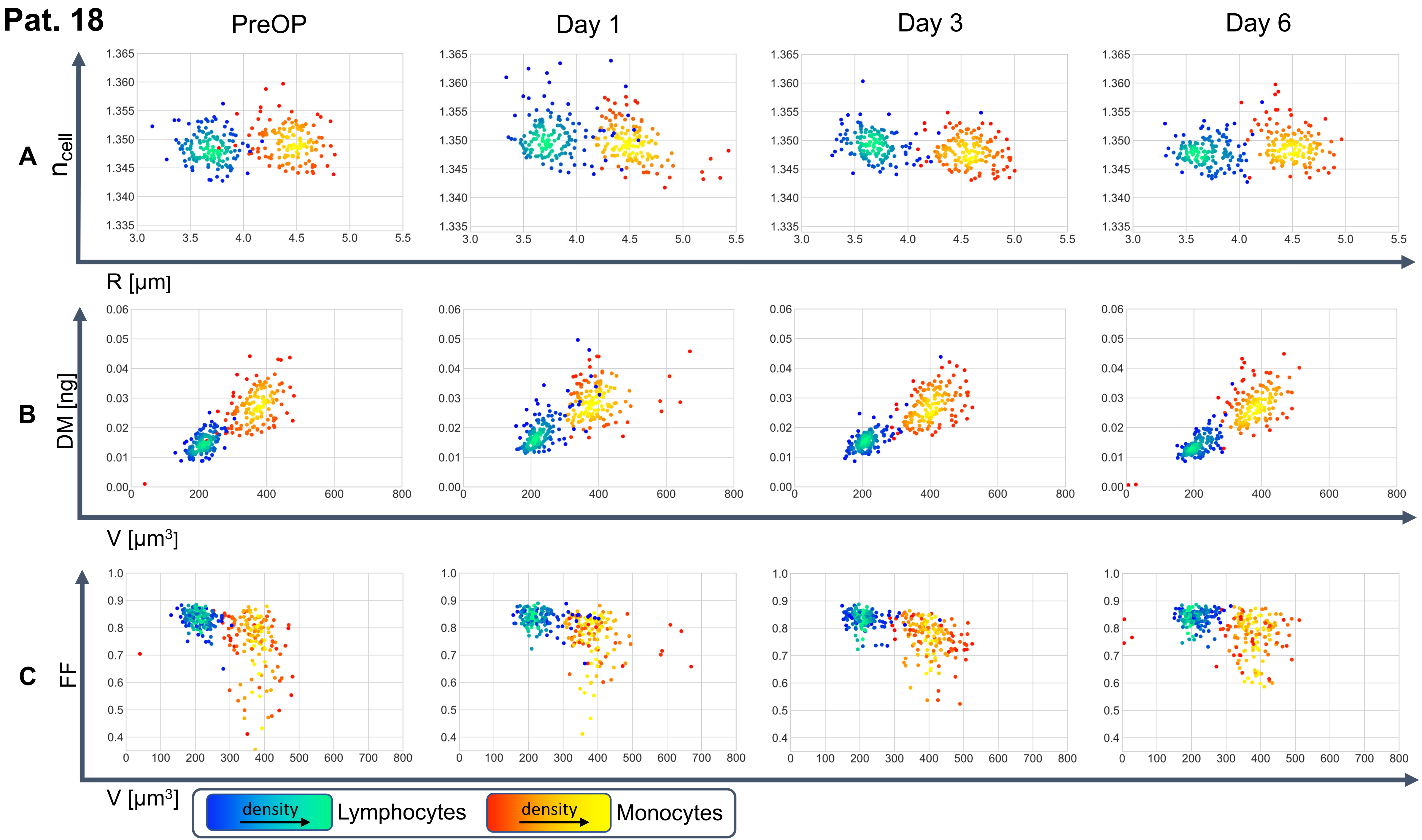

Pat. 18
PreOP
Day 1
Day 3
Day 6
A
n_{cell}
R [µm]
B
DM [ng]
V [µm³]
C
FF
V [µm³]
density
Lymphocytes
density
Monocytes

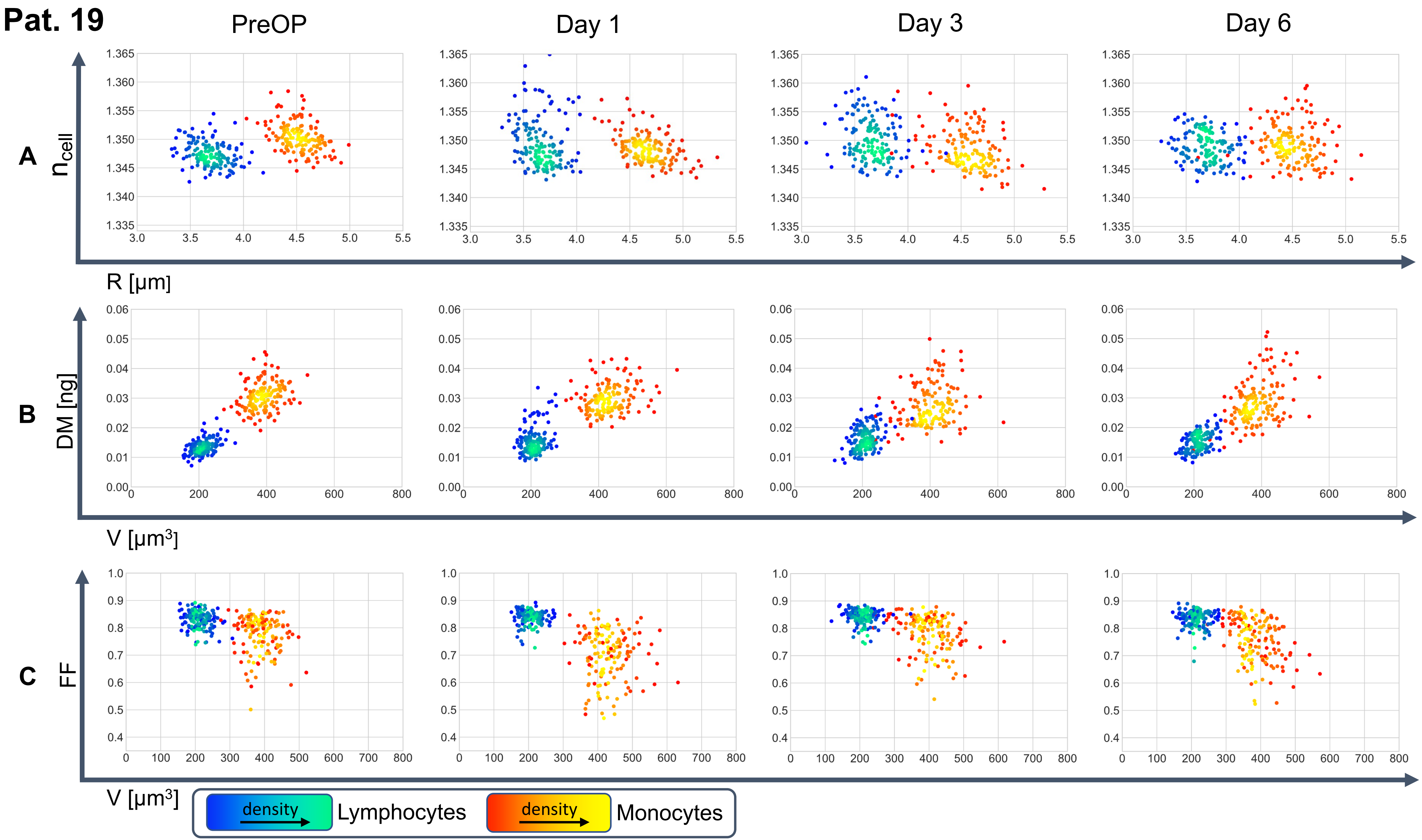
Pat. 19
PreOP
Day 1
Day 3
Day 6
A
n_cell
R [μm]
B
DM [ng]
V [μm³]
C
FF
V [μm³]
density
Lymphocytes
density
Monocytes

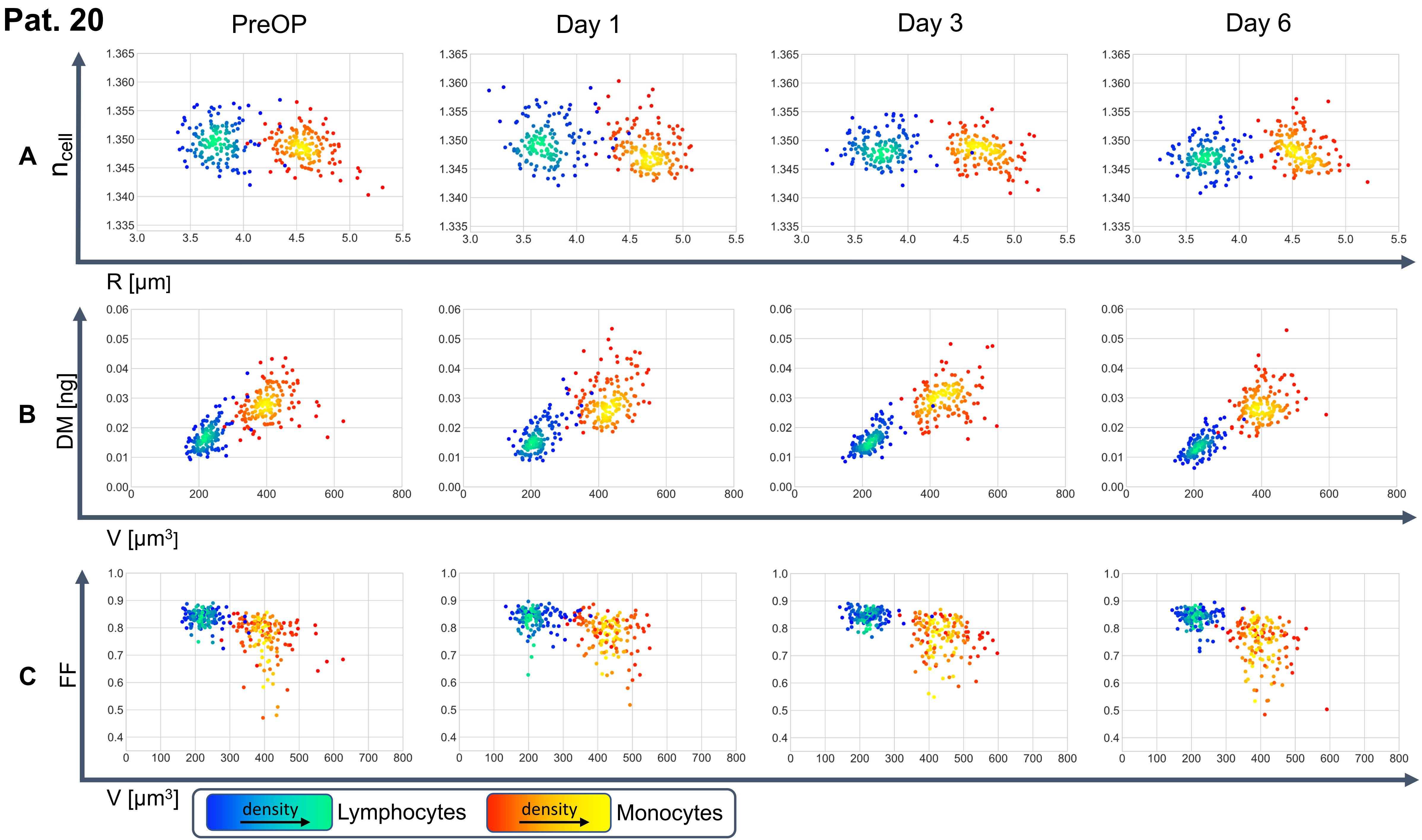

Pat. 20
PreOP
Day 1
Day 3
Day 6
A
n_{cell}
R [µm]
B
DM [ng]
V [µm³]
C
FF
V [µm³]
density
Lymphocytes
density
Monocytes

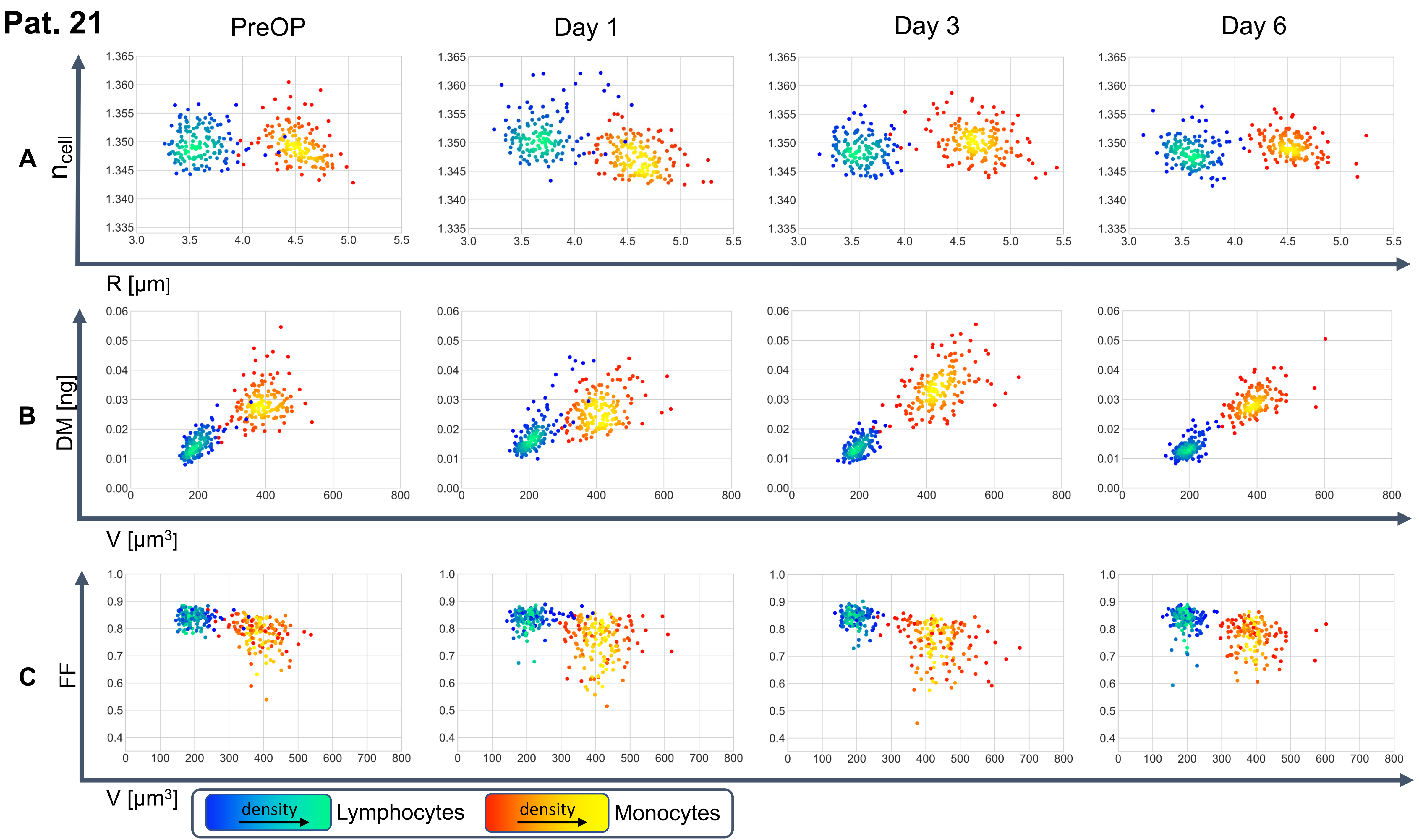

Pat. 21
PreOP
Day 1
Day 3
Day 6
A
n_cell
R [µm]
B
DM [ng]
V [µm³]
C
FF
V [µm³]
density
Lymphocytes
density
Monocytes

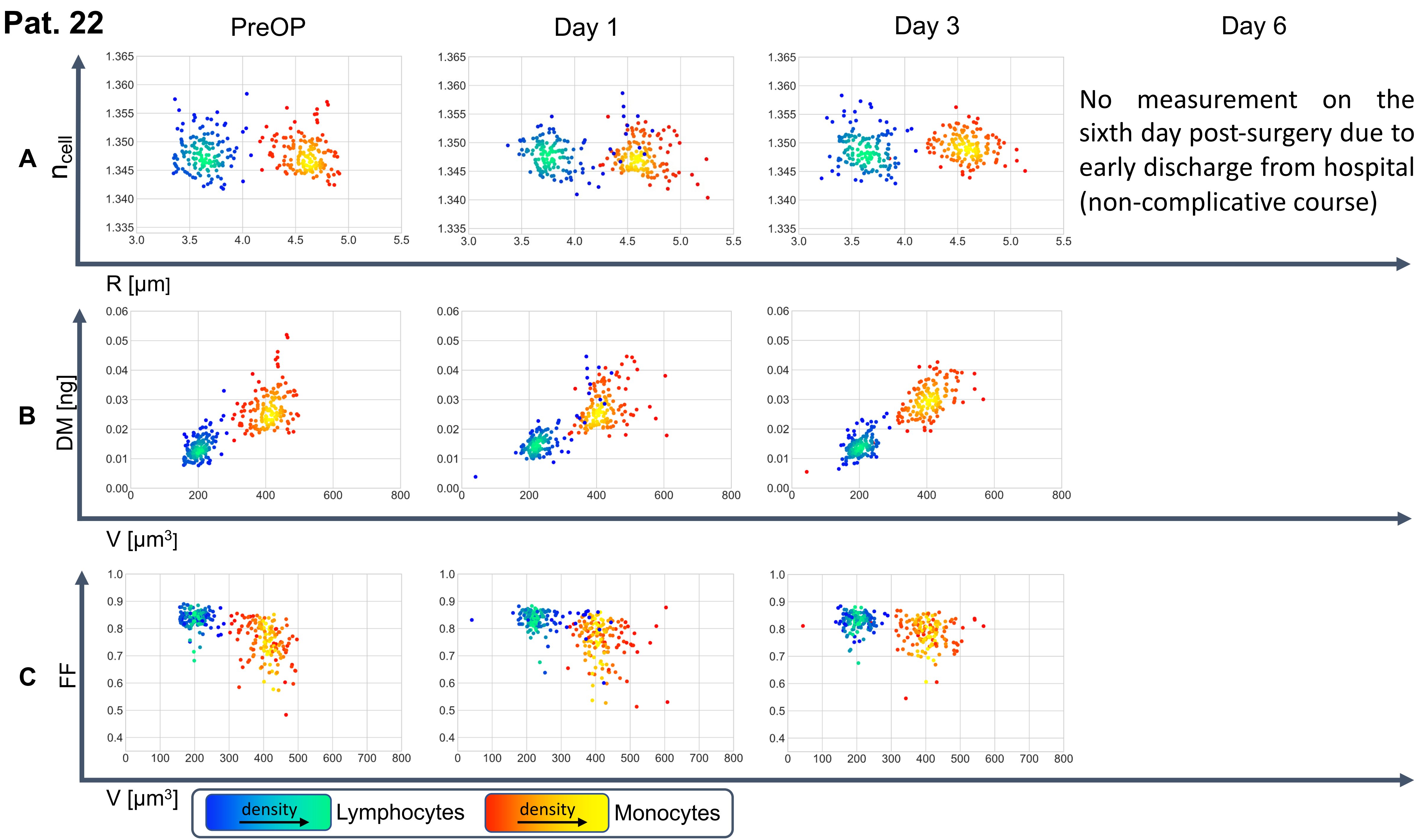
Pat. 22
PreOP
Day 1
Day 3
Day 6
No measurement on the sixth day post-surgery due to early discharge from hospital (non-complicative course)
A
B
C
n cell
R [µm]
DM [ng]
V [µm³]
FF
V [µm³]
density
Lymphocytes
density
Monocytes

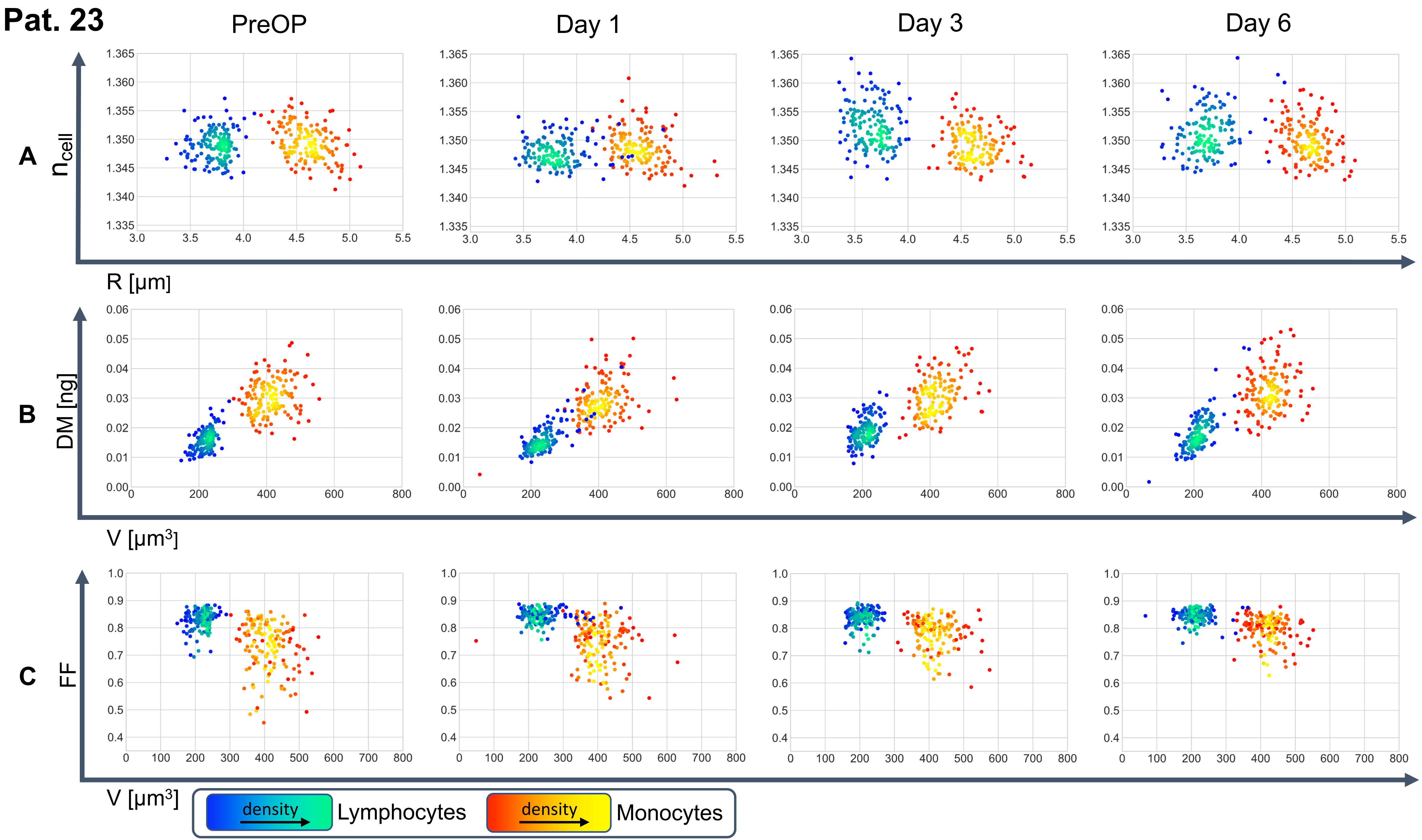
Pat. 23
PreOP
Day 1
Day 3
Day 6
A
n_{cell}
R [µm]
B
DM [ng]
V [µm³]
C
FF
V [µm³]
density
Lymphocytes
density
Monocytes

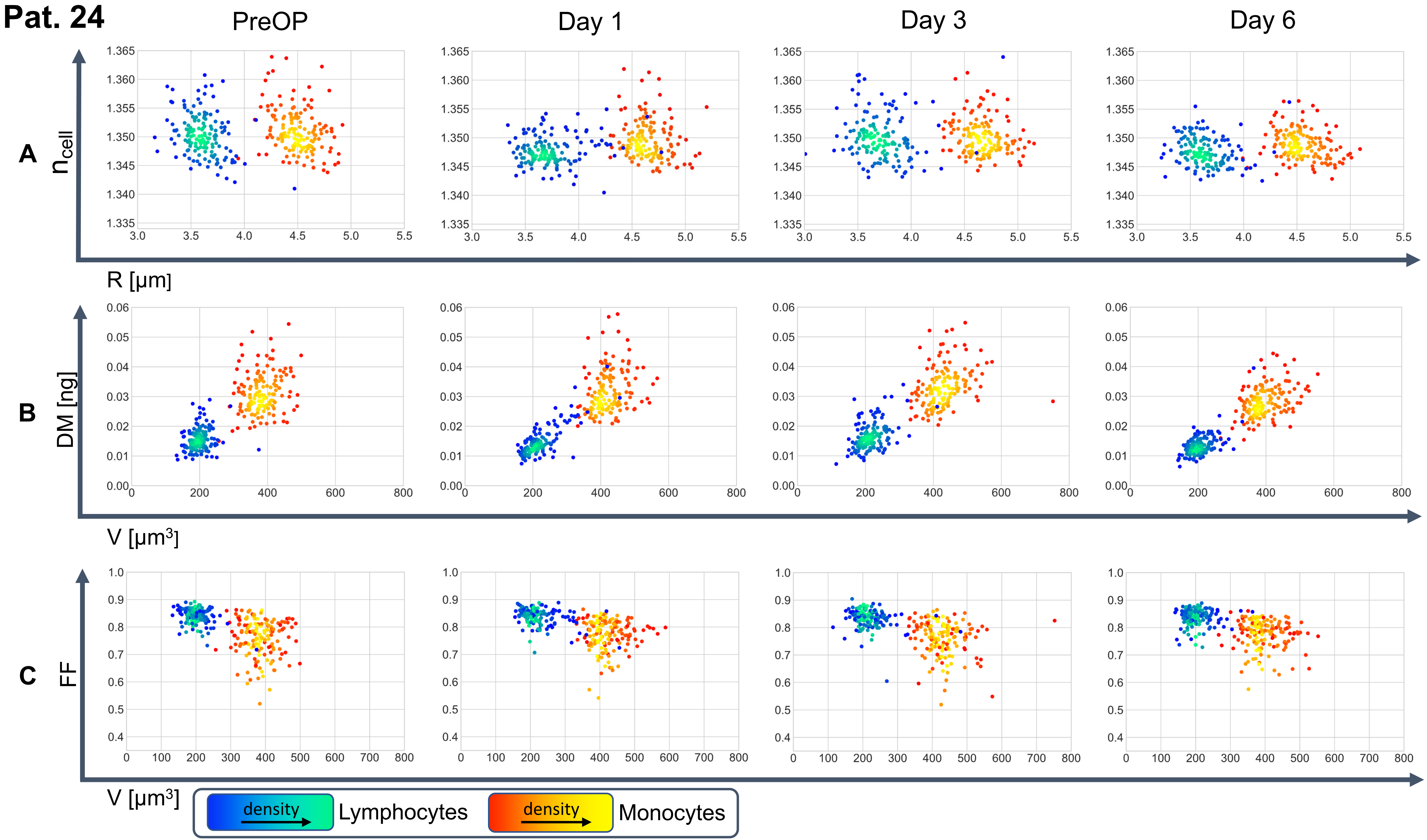

Pat. 24
PreOP
Day 1
Day 3
Day 6
A
n_{cell}
R [µm]
B
DM [ng]
V [µm³]
C
FF
V [µm³]
density
Lymphocytes
density
Monocytes

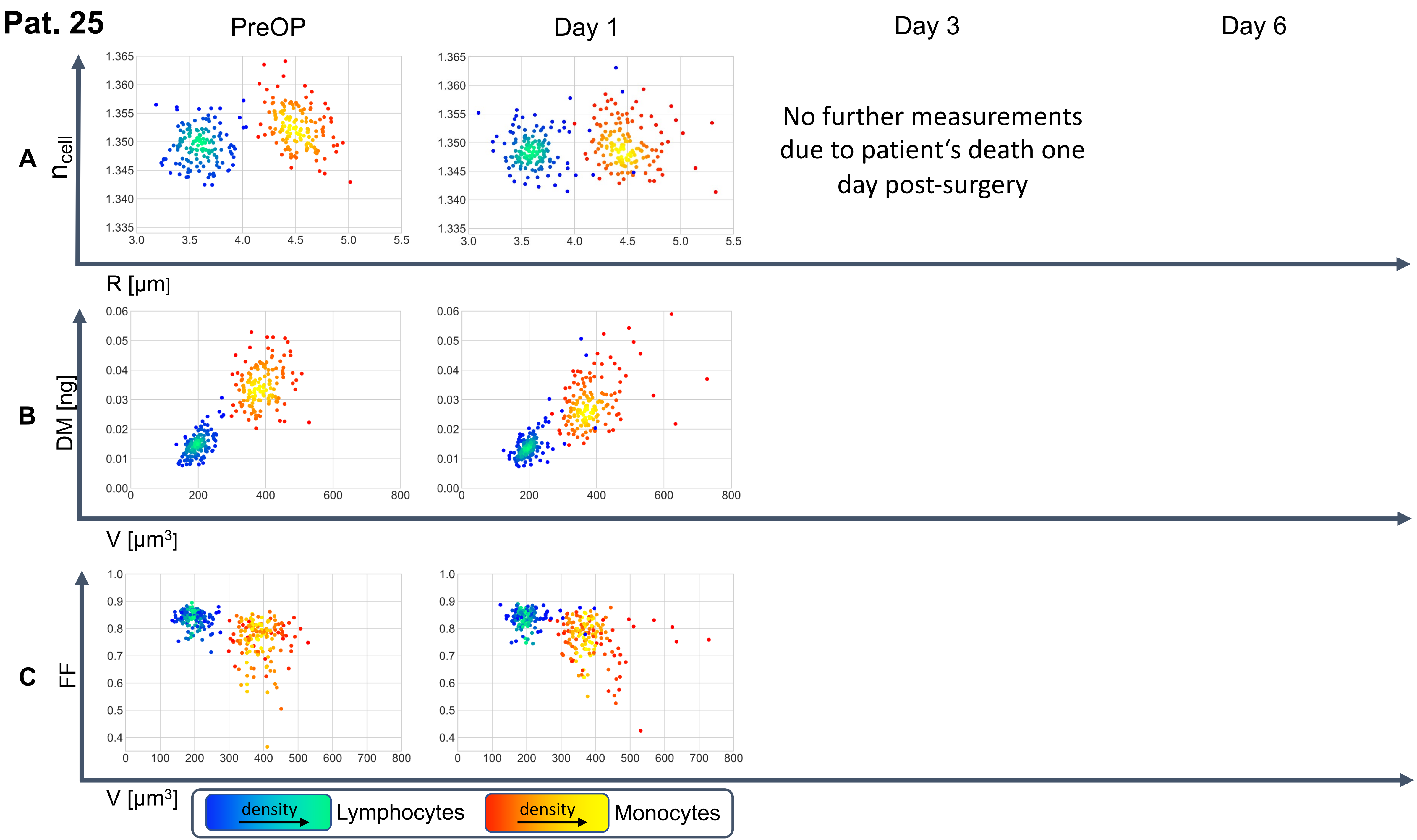
Pat. 25
PreOP
Day 1
Day 3
Day 6
No further measurements due to patient‘s death one day post-surgery
A
n_cell
R [µm]
B
DM [ng]
V [µm³]
C
FF
V [µm³]
density
Lymphocytes
density
Monocytes
