# Supplementary material for: Digital Holographic Microscopy for Label-Free Detection of Leukocyte Alternations Associated with Perioperative Inflammation after Cardiac Surgery
Source: Cells. 2022 Feb 21;11(4):755. doi: 10.3390/cells11040755 (PMC8869820; doi:10.3390/cells11040755)

Supplementary Figure S7

P values presented in form of a heat map. Bivariate correlations between changes of DHM data, flow cytometric parameters and epinephrine dose between timepoints PreOP and d1.

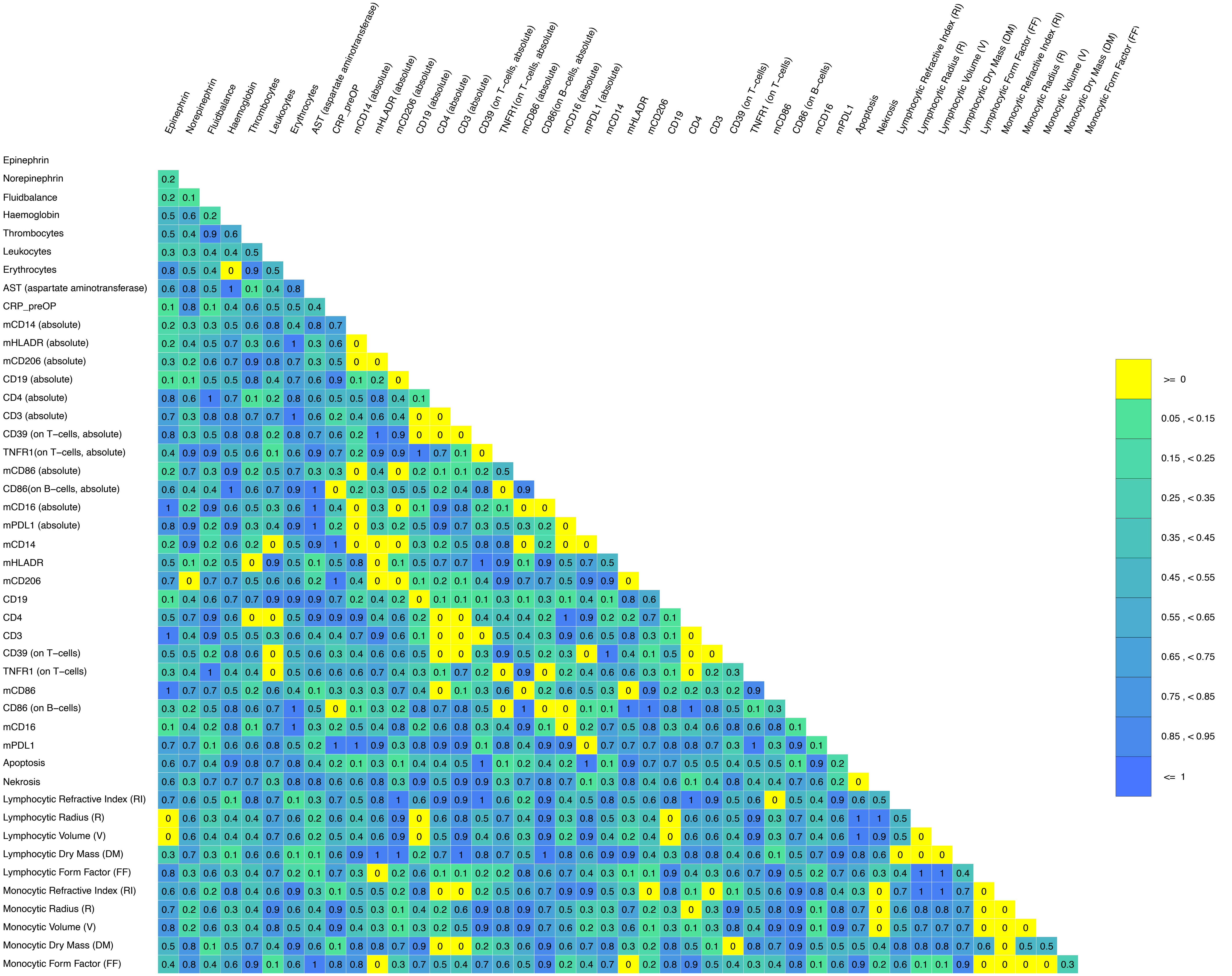

Supplementary Figure S8

Correlation coefficients presented in a heat map. Bivariate correlations between changes of DHM data, flow cytometric parameters and epinephrine dose between timepoints PreOP and d1.

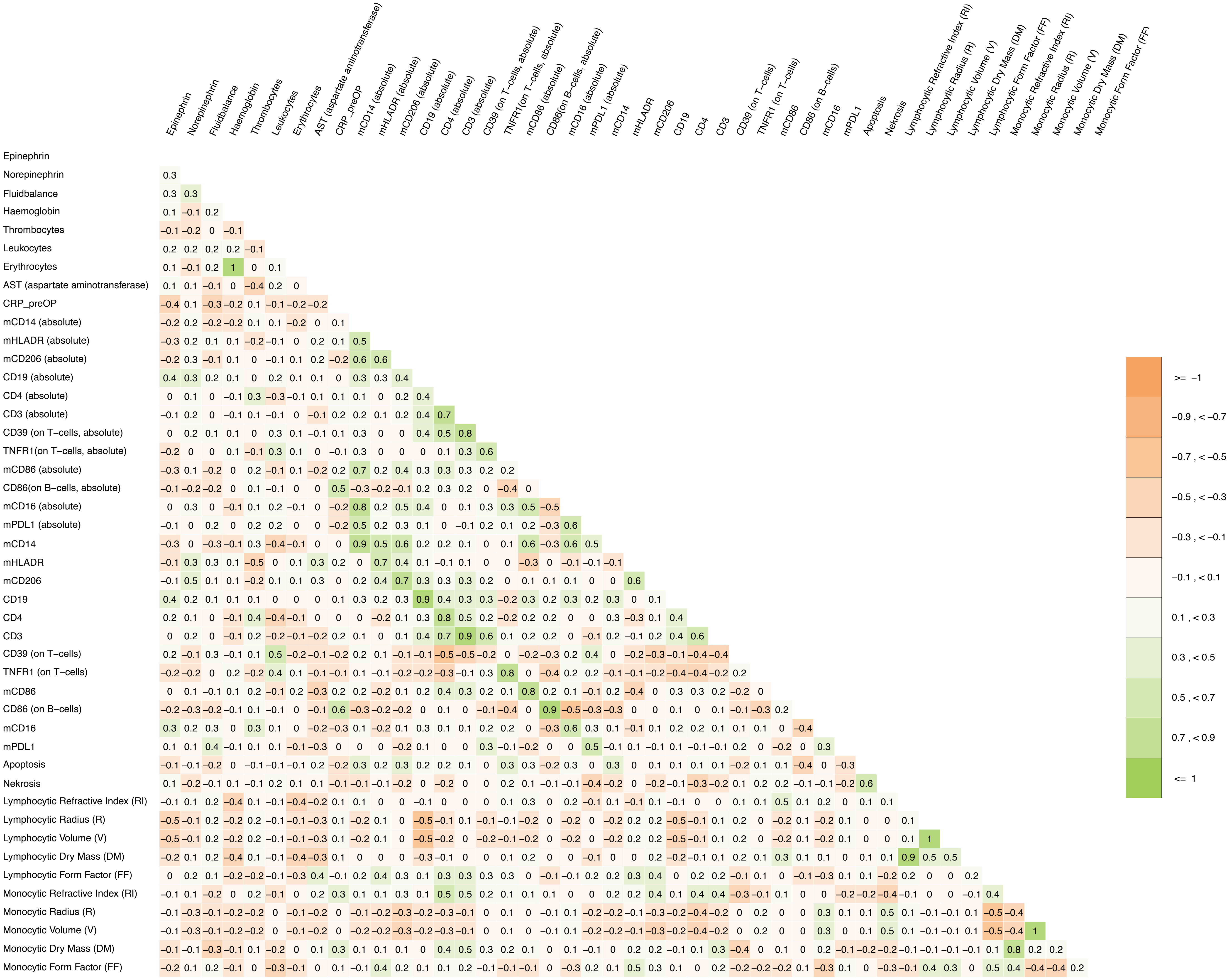

Supplement: Supplementary file 1 [file cells-11-00755-s001.zip › Supplement_S4.pdf]
